# Design, synthesis and antiproliferative screening of newly synthesized coumarin-acrylamide hybrids as potential cytotoxic and apoptosis inducing agents†

**DOI:** 10.1039/d3ra06644d

**Published:** 2023-11-06

**Authors:** Hany M. Abd El-Lateef, Lina M. A. Abdel Ghany, Rasha Mohammed Saleem, Ali Hassan Ahmed Maghrabi, Maryam Abdulrahman Yahya Alahdal, Eman Hussain Khalifa Ali, Botros Y. Beshay, Islam Zaki, Reham E. Masoud

**Affiliations:** a Department of Chemistry, College of Science, King Faisal University Al-Ahsa 31982 Saudi Arabia hmahmed@kfu.edu.sa; b Department of Chemistry, Faculty of Science, Sohag University Sohag 82524 Egypt; c Pharmaceutical Chemistry Department, College of Pharmaceutical Sciences and Drug Manufacturing, Misr University for Science and Technology 6th of October City Egypt; d Department of Laboratory Medicine, Faculty of Applied Medical Sciences, Al-Baha University Al-Baha 65431 Saudi Arabia; e Department of Biology, Faculty of Applied Science, Umm Al-Qura University Makkah 24381 Saudi Arabia; f Department of Biology, Faculty of Applied Science, Umm Al-Qura University Makkah 24381 Saudi Arabia; g Department of Laboratory Medicine, Faculty of Applied Medical Sciences, Albaha University Saudi Arabia; h Pharmaceutical Sciences (Pharmaceutical Chemistry) Department, College of Pharmacy, Arab Academy for Science, Technology and Maritime Transport Alexandria Egypt; i Pharmaceutical Organic Chemistry Department, Faculty of Pharmacy, Port Said University Port Said Egypt Eslam.Zaki@pharm.psu.edu.eg; j Clinical Pharmacology Department, Faculty of Medicine, Port Said University Port Said 42526 Egypt

## Abstract

On the basis of the observed biological activity of coumarin and acrylamide derivatives, a new set of coumarin-acrylamide-CA-4 hybrids was designed and synthesized. These compounds were investigated for their cytotoxic activity against cancerous human liver cell line HepG2 cells using 5-fluorouracil (5-FU) as a reference drug. Compound 6e had promising antiproliferative activity with an IC_50_ value of 1.88 μM against HepG2 cells compared to 5-FU (IC_50_ = 7.18 μM). The results of β-tubulin polymerization inhibition indicated that coumarin-acrylamide derivative 6e was the most active, with a percentage inhibition value of 84.34% compared to podophyllotoxin (88.19% β-tubulin inhibition). Moreover, the active coumarin-acrylamide molecule 6e exerted cell cycle cession at the G2/M phase stage of HepG2 cells. In addition, this compound produced a 15.24-fold increase in apoptotic cell induction compared to no-treatment control. These observations were supported by histopathological studies of liver sections. The conducted docking studies illustrated that 6e is perfectly positioned within the tubulin colchicine binding site, indicating a significant interaction that may underlie its potent tubulin inhibitory activity. The main objective of the study was to develop new potent anticancer compounds that might be further optimized to prevent the progression of cancer disease.

## Abstract 2

A sequence of novel coumarin-acrylamide hybrids has been synthesized and evaluated for their *in vitro* cytotoxicity against HepG2 liver cancer cells.

## Graphical abstract

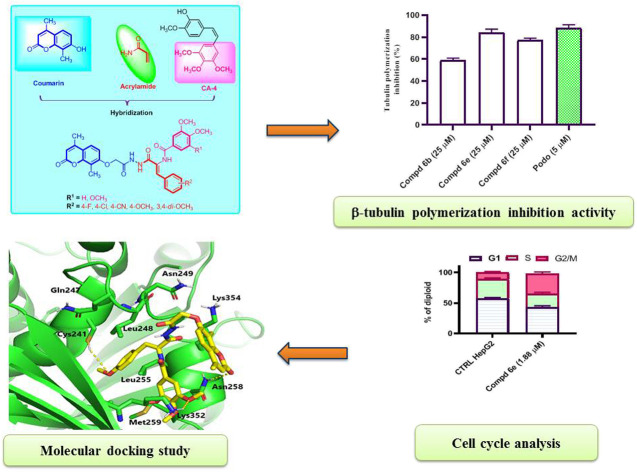

## 1. Introduction

Cancer stands as the world's foremost contributor to untimely fatalities caused by non-communicable ailments.^1^ It represents an unbridled proliferation of irregular cells attributed to malfunctions within the apoptotic process.^2–4^ Hepatocellular carcinoma (HCC), an aggressive tumor, emerges as the predominant form of primary liver malignancy. HCC stands as the second primary cause of cancer-related deaths, characterized by its grim prognosis and formidable resistance to traditional chemotherapy treatments.^5^ As per the World Health Organization, liver cancer ranks as the most prevalent malignancy among the Egyptian population, boasting incidence and mortality rates of 19.7% and 29.4%, respectively.^6^

Coumarins, characterized by their fundamental structural core of benzopyrone, have attracted significant attention in the scientific community due to their abundant sources, easy synthesis methods, and diverse pharmacological properties.^7–9^ Notably, coumarins exhibit impressive anti-tumor capabilities through a variety of mechanisms, including carbonic anhydrase inhibition, targeting of PI3K/Akt/mTOR signaling pathways, activation of cell apoptosis proteins, suppression of tumor multidrug resistance, interference with microtubule polymerization, regulation of reactive oxygen species, and inhibition of tumor angiogenesis.^9,10^ Whether obtained naturally from different plants or created through modifications to the core coumarin structure, both forms of coumarins have demonstrated their ability, in laboratory experiments, to effectively inhibit the growth and proliferation of tumor cells at low concentrations without harming normal cells.^9^ This underscores coumarins' potential as a promising class of highly selective anti-tumor drugs.

The acrylate component serves as a prevalent structural framework found in a wide array of both natural and artificially synthesized small molecules that exhibit a wide range of intriguing biological properties.^11,12^ It has been documented to manifest various biomedical effects, including its potential as an anticancer agent.^13^ The exploration of acrylate-based compounds has brought forth novel molecules with the capacity to act as anticancer agents, involving diverse mechanisms such as the inhibition of β-tubulin and protein kinases.^14,15^ These discoveries indicate that the acrylate pharmacophore presents a promising molecular foundation for further adaptations aimed at developing more potent candidates for anticancer drugs.^16^

Furthermore, combretastatin A-4 (CA-4) stands out as the most prevalent member within the combretastatin family, originally derived from the African *Combretum caffrum* tree.^17–19^ CA-4 displays potent antimitotic properties by binding to the colchicine binding site, with the dimethoxybenzene portion, along with other elements of the CA-4 ring, playing a pivotal role in enhancing binding affinity. CA-4 advanced to phase II and phase III stages during clinical trials.^20,21^ However, CA-4 is plagued by several pharmacokinetic drawbacks, including limited water solubility,^22–24^ as well as a short plasma half-life and susceptibility to isomerization from the active *cis* form to the inactive *trans* form under *in vivo* conditions.

**Fig. 1:**
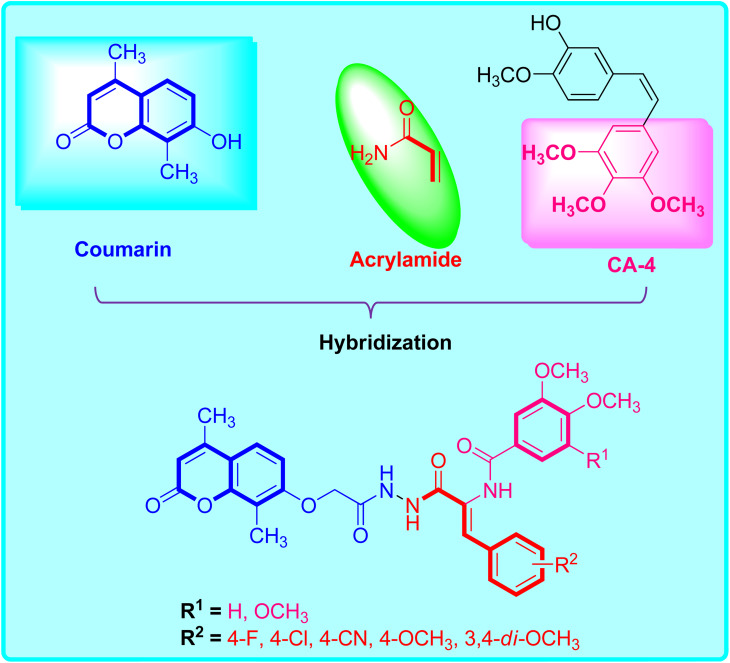
Molecular design of the prepared coumarin-acrylamide hybrids 5 and 6a–f.

In light of these discoveries, our objective is to devise innovative antitubulin inhibitors, employing a hybrid structure-based approach as an effective strategy for creating novel drug candidates.^8,25^ This initiative aims to address the escalating prevalence of resistance to current therapeutic remedies. In this endeavor, we have amalgamated 7-substituted coumarin, the acrylate framework, and the trimethoxybenzene component from CA-4 (as depicted in Fig. 1) into a unified molecule with the goal of enhancing their capacity to inhibit microtubule polymerization. Furthermore, we have implemented various substitution patterns on both the central phenyl and the distally substituted trimethoxybenzene ring to investigate their influence on tubulin-binding and inhibitory activity akin to colchicine. The underlying purpose of this design is to probe the potential antiproliferative effects of these compounds on the HepG2 liver cancer cell line.

## 2. Results and discussion

### 2.1. Chemistry

**Scheme 1:**
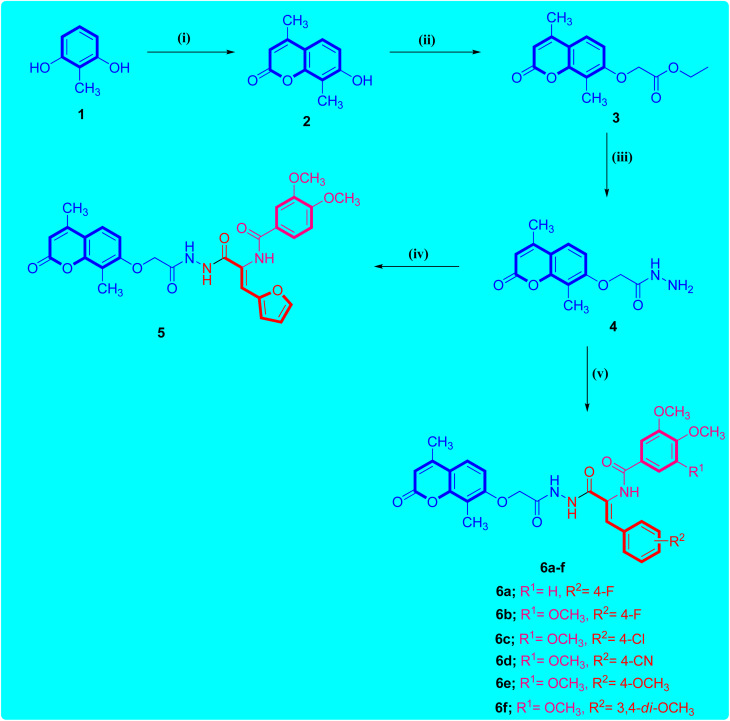
Schematic representation of the route adopted for the synthesis of coumarin–acrylamide hybrids 5 and 6a–f. Reagents: (i) CH_3_COCH_2_COOC_2_H_5_, conc. H_2_SO_4_, stirring overnight at rt, yield = 91%; (ii) ClCH_2_COOEt, K_2_CO_3_, acetone, reflux 48 h, yield = 86%; (iii) NH_2_NH_2_·H_2_O, EtOH, reflux 6 h, yield = 78%; (iv) ethyl 2-(3,4-dimethoxybenzamido)-3-(furan-2-yl)acrylate, EtOH, AcOH, reflux 6 h, yield = 71%; (v) ethyl 2-(arylamido)-3-arylacrylate, EtOH, AcOH, reflux 6–8 h, yield: 59–74%.

Scheme 1 illustrates the adopted synthetic pathway for the target coumarin-acrylamide compounds 5 and 6a–f. The key hydrazide intermediate 4 was synthesized by reported by *O*-alkylation of 4,8-dimethyl-7-hydroxycoumarin 2 with ethyl 2-chloroacetate in the presence of potassium carbonate to give coumarin ethyl acetate ester 3 that underwent hydrazinolysis with hydrazine hydrate in pure ethanol to afford the key coumarin acetohydrazide intermediate 4 in good yield.^26^

The reaction of the key acetohydrazide intermediate 4 with the respective aryl acrylate ethyl ester in absolute ethanol and a catalytic amount of glacial acetic acid promotes the reaction to afford the target coumarin-aryl acrylamide hybrids 5 and 6a–f. The structures of the obtained coumarin-aryl acrylamide derivatives 5 and 6a–f were verified by spectroscopic methods and elemental microanalytical data. For instance, the ^1^H-NMR spectrum of coumarin-3-(4-chlorophenyl)acrylamide derivative 6c as an example showed one singlet (*δ* 10.26) and two doublets (*δ* 10.40 and 9.79) for the three amide protons. The protons of the 4,8-dimethylcoumarin moiety appeared as two doublets for one proton each at *δ* 7.73 and 7.02 ppm with a coupling constant of 8.3 Hz and singlet for one proton at *δ* 6.98 ppm. In addition, the protons of the 3-(4-chlorophenyl)acrylamide function appeared as two doublets for two protons each with a coupling constant of 8.1 Hz. Besides, the presence of a doublet (*δ* 7.38 ppm) with a coupling constant of 7.0 Hz was assigned to olefin proton of the acrylamide moiety. Additionally, the methoxy protons of the 3,4,5-trimethoxybenzamide group appeared as two singlets at *δ* 3.85 and 3.74 ppm for two methoxy and one methoxy groups, respectively. Furthermore, the signal observed at *δ* 4.80 ppm was assigned to methylene (OCH_2_) protons in the compound. Additionally, the two methyl protons appeared as two singlets at *δ* 2.37 and 2.08 ppm for three protons each.

The ^13^C-NMR spectrum of coumarin-3-(4-chlrophenyl)acrylamide compound 6c showed three peaks at *δ* 166.69–164.74 ppm attributed to the carbonyl carbon of triamide functions. The signal observed at *δ* 66.64 ppm was assigned to methylene (OCH_2_) carbon. In addition, the methoxy carbons of the 3,4,5-trimethoxybenzamide group appeared at *δ* 60.57 and 56.54 ppm for two methoxy carbons and one methoxy carbon, respectively. Moreover, the two methyl carbons resonated at *δ* 15.40 and 13.40 ppm. Finally, the remaining aromatic and olefinic carbons resonated at around *δ* 161.63–101.82 ppm.

### 2.2. Biology

#### 2.2.1. *In vitro* cytotoxic activity against HepG2 cancerous human liver cell line

The prepared coumarin-aryl acrylamide hybrids 5 and 6a–f were evaluated for their *in vitro* cytotoxic activity against the HepG2 cancerous human liver cell line through the use of a colorimetric MTT assay. 5-Fluorouracil (5-FU) was utilized as a positive standard because it is the first line of treatment for hepatocellular carcinoma.^27^ Moreover, the most active coumarin-3-(4-methoxyphenyl)acrylamide derivative 6e was tested on normal human liver cell line (HL-7702) for determining its cytotoxic impact in comparison with 5-FU. The results revealed that the tested coumarin-3-(4-methoxyphenyl)acrylamide derivative 6e suppressed the growth of HL-7702 cells with IC_50_ value less than that of 5-FU suggesting that the synthesized coumarin–acrylamide molecule exhibited a lower cytotoxic effect on normal liver cells compared to HepG2 liver carcinoma cells. The *in vitro* cytotoxic results against HepG2 cells are summarized in Table 1 as IC_50_ values in μM concentration. Examination of the obtained cytotoxicity results against HepG2 cancerous human liver cells revealed that substitution on the coumarin acetohydrazinyl scaffold with different aryl acrylamide moieties gave rise to remarkable cytotoxic activity with IC_50_ values between 1.88 and 27.04 μM. It was found that the presence of an electron-donating methoxy (OCH_3_) substituent on the aromatic ring as in compounds 6e and 6f resulted in higher activity compared with an electron-withdrawing function on the aromatic ring as can be seen in compounds 6b and 6c. It could also be observed that coumarin-3-(4-fluorophenyl)acrylamide bearing 3,4-dimethoxybenzamide moiety (6a) showed moderate cytotoxic activity (IC_50_ = 27.04 μM) and it was the least active compared with other coumarin-acrylamide derivatives. On the other hand, the coumarin-3-(4-methoxyphenyl)acrylamide possessing a trimethoxybenzamide group (6e) was the most potent compound against HepG2 cells exhibiting an IC_50_ value of 1.88 μM compared with 5-FU (IC_50_ of 7.18 μM).

**Table 1:** Cytotoxicity (IC_50_, μM) of the newly synthesized coumarin-arylacrylamide hybrids 5 and 6a–f; values represent the mean IC_50_ values ± SEM (*n* = 3) for each hybrid^a^

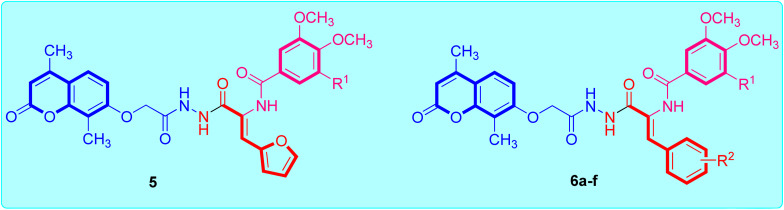				
Compound	R^1^	R^2^	IC_50_ value (μM)	
			HepG2	HL-7702
5	H	—	13.18 ± 1.12	NT
6a	H	4-F	27.04 ± 1.43	NT
6b	OCH_3_	4-F	6.77 ± 0.83	NT
6c	OCH_3_	4-Cl	18.85 ± 1.27	NT
6d	OCH_3_	4-CN	8.77 ± 0.99	NT
6e	OCH_3_	4-OCH_3_	1.88 ± 0.27	51.46 ± 0.38
6f	OCH_3_	3,4-*di*-OCH_3_	4.14 ± 0.62	NT
5-FU	—	—	7.18 ± 0.14	35.11 ± 0.19

a NT: not tested.

#### 2.2.2. β-Tubulin inhibition assay

**Fig. 2:**
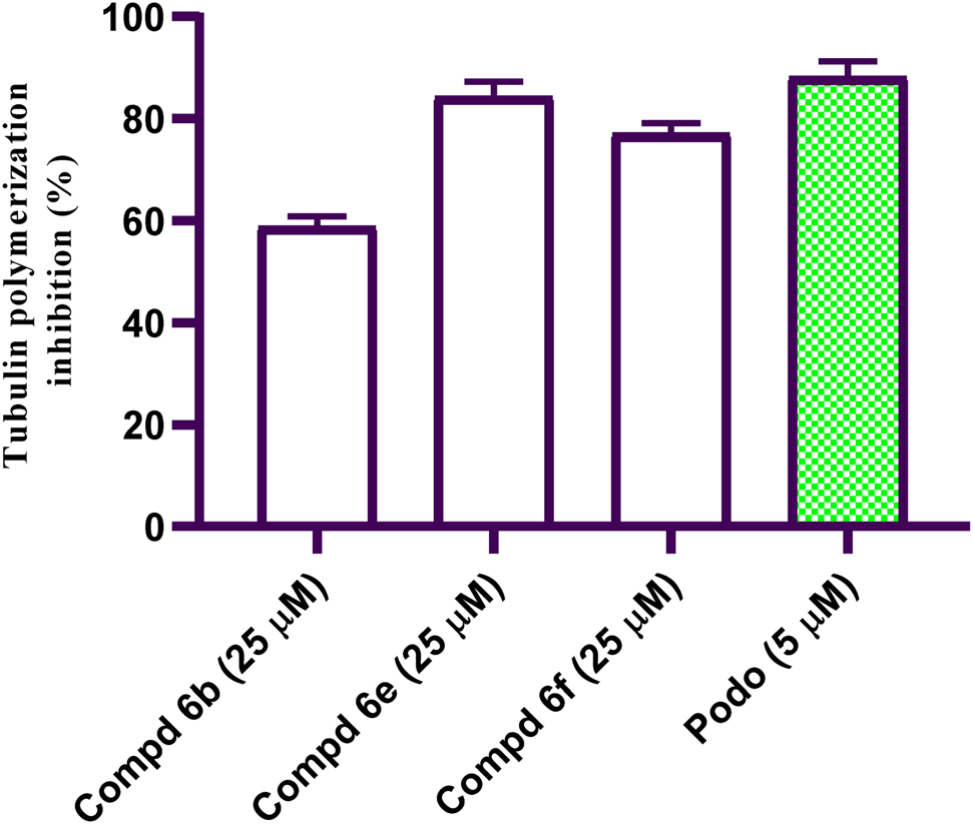
Results of the β-tubulin polymerization inhibition assay as a percent inhibition value for active coumarin-acrylamide molecules 6b, 6e, and 6f compared to podophyllotoxin.

Excessive β-tubulin polymerization has been associated with tumor progression and a poor prognosis in most solid tumors, including liver malignancy.^28^ Targeting β-tubulin polymerization is one of the most effective ways to develop new cancer regimens.^29^ Active coumarin-acrylamide hybrids 6b, 6e, and 6f were tested in a β-tubulin inhibition assay according to a reported protocol^30^ using podophyllotoxin as the reference standard. The results of the β-tubulin inhibition assay for selected coumarin-acrylamide hybrids are depicted as percentage inhibition values (%) and are presented in Fig. 2. Results revealed that coumarin-3-(4-methoxyphenyl)acrylamide derivative 6e elicited comparable β-tubulin inhibition activity to that of podophyllotoxin, with a percent inhibition value of 84.34% compared to podophyllotoxin (88.19% inhibition). On the other hand, coumarin-3-(3,4-dimethoxyphenyl)acrylamide 6f was slightly less potent than podophyllotoxin, with a percent inhibition value of 77.15%, while coumarin-3-(4-fluorphenyl)acrylamide 6b (58.92% inhibition) elicited nearly half the inhibitory activity of podophyllotoxin against β-tubulin polymerization. The results confirmed that the antiproliferative activity of coumarin-acrylamide hybrids 6e and 6f may be mediated by the inhibition of β-tubulin polymerization.

#### 2.2.3. Cell cycle analysis

**Fig. 3:**
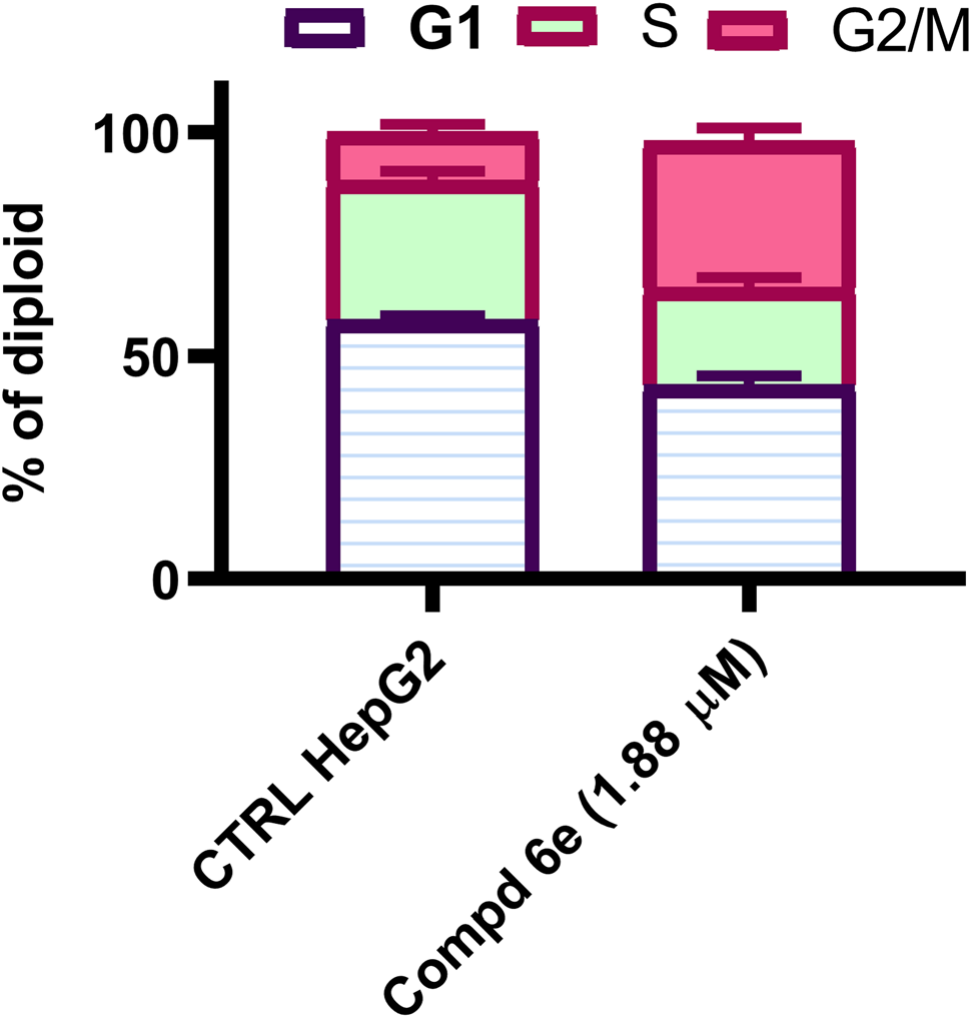
Results of the cell cycle assay as percentage cell count induced by active coumarin-acrylamide molecule 6e in each phase of the cellular cycle of HepG2 cells.

Most cytotoxic pharmaceuticals are known to have an antiproliferative impact by interrupting the cellular cycle at certain phase points.^31^ To characterize the growth inhibition induced by the coumarin-acrylamide hybrids, we examined the impact of the most potent coumarin-aryl acrylamide hybrid 6e on cell cycle advancement in order to define the point at which the stoppage of cell cycle arrest develops in HepG2 cells. The impact on cell cycle distribution was assessed by a DNA flow cytometric analysis, upon incubation of HepG2 cells with coumarin-acrylamide hybrid 6e at the IC_50_ concentration for 48 h. As shown in the results in Fig. 3, treatment of HepG2 cells with coumarin-acrylamide derivative 6e resulted in a significant decrease in cell population at the G1 and S phases, from 58.03 and 31.11% for the untreated control to 43.46 and 21.62%, respectively, for the cells treated with coumarin-acrylamide compound 6e. On the other hand, the cellular population rose in the G2/M phase from 10.86% for untreated HepG2 cells to 34.92% for HepG2 cells treated with active hybrid 6e. These results suggested that the coumarin-acrylamide compound 6e stopped HepG2 cells in the G2/M phase.

#### 2.2.4. Annexin V assay

**Fig. 4:**
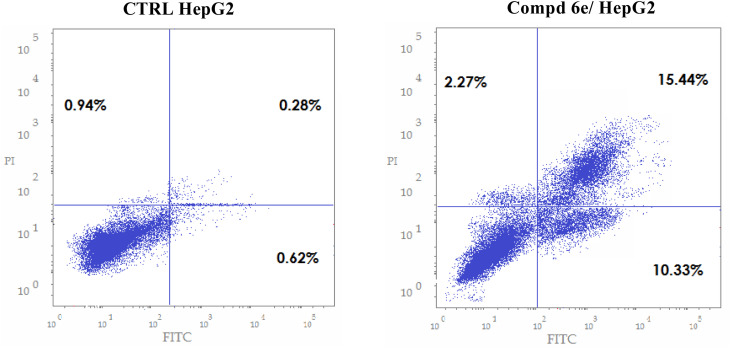
Results of the Annexin V-FITC/PI assay of the effect of the most active coumarin-acrylamide molecule 6e on cancerous human liver HepG2 cells.

Apoptosis, also called programmed cell death, involves several morphological and biochemical events.^32^ Apoptosis analysis with propidium iodide (PI) solution and protein Annexin V with HepG2 cells was conducted to assess the efficacy of the coumarin-acrylamide hybrids to cause cancer cell apoptosis. HepG2 cells were treated with the IC_50_ concentration (1.88 μM) of coumarin-acrylamide derivative 6e for 48 h. The data are shown as fluorescent-activated cell filter flow cytometry panels (Fig. 4), with PI staining in the *Y* direction of the graph and fluorochrome Annexin V in the *X* direction of the graph. It can be seen in Fig. 4 that the percentage of apoptosis rose from 1.84% for control untreated HepG2 cells to 28.04% in cells treated with coumarin-acrylamide hybrid 6e. It is noteworthy that the proportion of cells in the early apoptosis state after treatment with coumarin-acrylamide molecule 6e rose by 16.67-fold in comparison with the untreated HepG2 control. In parallel, the cellular population of the late apoptosis cells after treatment with coumarin-acrylamide hybrid 6e went up by 55.14-fold as compared with control untreated HepG2 cells. Furthermore, as seen in Fig. 4, the percentage of late apoptosis (15.44%) is greater than that of early apoptosis (10.33%), indicating that the coumarin-acrylamide molecule 6e induces irreversible apoptosis.

#### 2.2.5. Histopathological investigation of liver sections

**Fig. 5:**
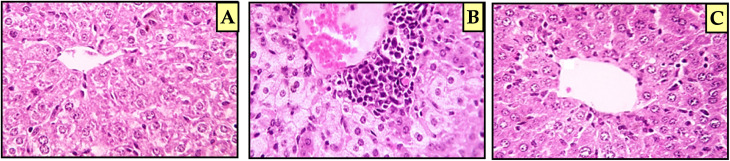
(A) Histopathological examination of the liver section from the control untreated mice group revealed normal histological structure of hepatic tissue. (B) The liver section of the EAC cell-bearing mice group showed distorted hepatocyte architecture with cytoplasmic vacuolization. (C) Liver section of EAC cell-bearing mice treated with coumarin-3-(4-methoxyphenyl)acrylamide 6e revealed mild damage to liver cells and less disruption of the hepatic cord.

The current histopathological characteristics appear to be consistent with the current *in vitro* findings. Histological examination of the liver of the control mice group demonstrated normal architecture, with normal liver lobules and hepatocyte cords disseminating from the central vein towards the hepatic lobule periphery. The hepatic cells are polygonal in shape and have one or two round nuclei (Fig. 5A). On the other hand, the liver section of the EAC cell-bearing mice group showed distorted hepatocellular architecture with swollen, degenerated, and necrotic hepatocytes and marked portal mononuclear cell infiltration, in addition to congestion in hepatoportal blood vessels (Fig. 5B). There was an improvement of histopathological characteristics after the administration of coumarin-3-(4-methoxyphenyl)acrylamide derivative 6e, as presented in Fig. 5C, with substantially better changes than that of the positive control group in the form of seemingly good parenchyma with mild focal pericentral mononuclear cell infiltration. Additionally, the liver section of the mice group given coumarin-3-(4-methoxyphenyl)acrylamide derivative 6e elicited less disruption of hepatic cords with a reduction in the number of liver cells with large nuclei. From these observations, we concluded that coumarin-3-(4-methoxyphenyl)acrylamide derivative 6e could have a significant potential therapeutic impact on liver tissue.

### 2.3. Molecular modeling study

**Fig. 6:**
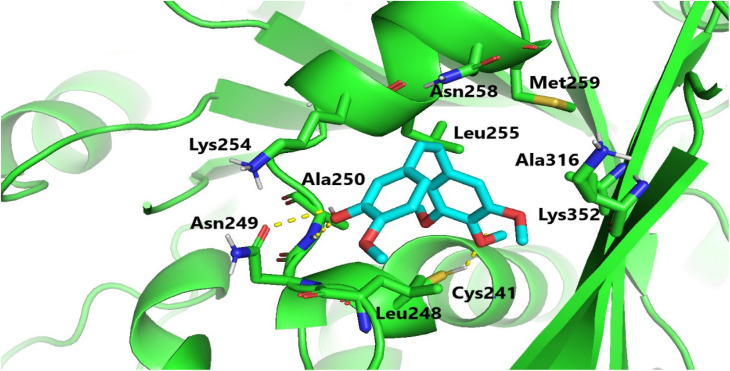
Binding configuration of CA-4 in tubulin's colchicine-binding pocket with highlighted hydrogen bonds.

**Fig. 7:**
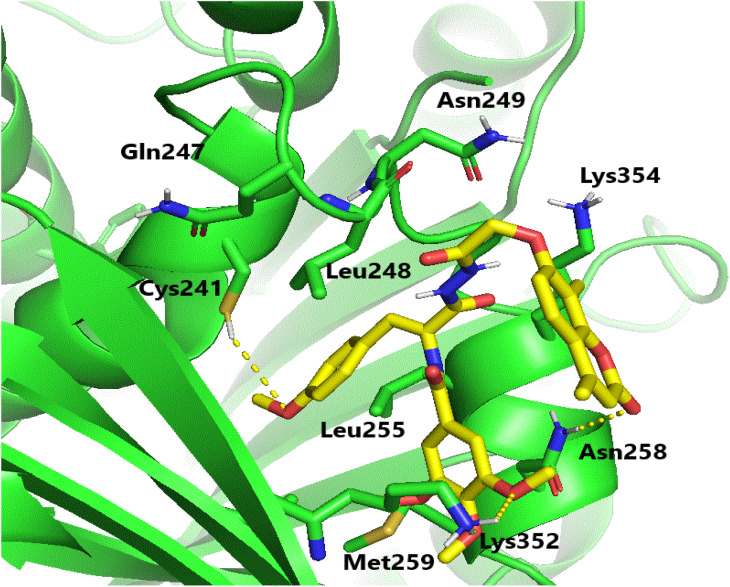
Binding configuration of active coumarin-acrylamide hybrid 6e in tubulin's colchicine-binding pocket with highlighted hydrogen bonds.

Molecular docking investigations were carried out to elucidate the exceptional cytotoxicity and tubulin inhibitory potency of the most potent compound, 6e, in comparison to CA-4. Interestingly, both CA-4 and compound 6e were aligned and positioned precisely within the colchicine binding site of tubulin, exhibiting binding free energies of −6.2 and −5.9 kcal mol^−1^, respectively. Furthermore, CA-4 and compound 6e (as depicted in Fig. 6 and 7) both established H-bonds with the pivotal amino acid residue Cys241. Regarding compound 6e, the *p*-methoxyphenyl acrylamide moiety formed the crucial H-bond with Cys241 through its methoxy functionality. Besides, it established strong hydrophobic contacts with Leu248, Ala250, Leu255, Lys352, and Ala316, anchoring the methoxyphenyl acrylamide moiety in the tubulin pocket. Furthermore, the trimethoxy benzene ring flanked the polar pocket formed by Asn258, Met259, and Lys352. Moreover, the coumarin scaffold formed an H-bond with Asn258 and had a hydrophobic interaction with Lys354.

It is worth noting that the formation of an H-bond with Cys241, along with the hydrophobic interactions involving the side chains of the aforementioned amino acid residues, as well as various polar interactions, significantly contributed to the protein-ligand binding process. These interactions may potentially explain the remarkable tubulin inhibitory activity observed for coumarin-acrylamide compound 6e.^33–35^

## 3. Conclusions

In this study, a new set of coumarin-acrylamide hybrids was designed and synthesized and their biological activities were evaluated as potent anti-tubulin activities. The results showed promising cytotoxic activity against the HepG2 cancerous human liver cell line. Among them, coumarin-3-(4-methoxyphenyl)acrylamide 6e (IC_50_ = 1.88 μM) and coumarin-3-(3,4-dimethoxyphenyl)acrylamide 6f (IC_50_ = 4.14 μM) were the most active against HepG2 cells, compared to 5-FU (IC_50_ = 7.18 μM). The results indicated that compound 6e showed good β-tubulin polymerization inhibitory potency with a percentage inhibition value of 84.34% compared to podophyllotoxin (88.19% polymerization inhibition). In addition, compound 6f showed moderate activity with a percent inhibition of 77.15%. Moreover, compound 6e exerted a significant increase in the level of HepG2 cells at the G2/M phase from 10.86% to 34.92%. Additionally, compound 6e demonstrated a significant increase in apoptotic cells in the late stage, from 0.28% to 15.44%. Furthermore, histopathological study of the EAC cell-bearing mice group treated with compound 6e revealed a mild improvement in histopathological characteristics. The outcomes of the docking studies imply that the anti-tubulin polymerization activity of the hybrid compound 6e, which combines coumarin and acrylamide moieties, may underlie its cytotoxic effects against the HepG2 liver cancer cell line. Notably, 6e with CA-4 exhibited precise alignment within the colchicine binding site of tubulin. Compound 6e formed a crucial H-bond with Cys241 and established strong hydrophobic contacts with several key amino acid residues, effectively anchoring itself within the tubulin pocket. These interactions, along with polar interactions, likely contribute to the outstanding tubulin inhibitory activity observed for compound 6e. Finally, coumarin-acrylamide molecule 6e has been identified as a new molecular scaffold that might be further modified to produce an intriguing chemotherapeutic candidate with potent β-tubulin polymerization inhibitory activity.

## 4. Experimental

### 4.1. Chemistry

#### 4.1.1. General

General details are in the ESI.†

#### 4.1.2. General procedure for the synthesis of 2-(4,8-dimethyl-2-oxo-2*H*-chromen-7-yloxy)acetohydrazide (4)

A mixture of the corresponding coumarin ethyl acetate ester 3 (1 g, 3.62 mmol) and hydrazine hydrate (0.58 g, 18.1 mol) in absolute ethanol (20 mL) was heated to reflux for 6 h, concentrated to half its volume and the precipitate obtained was filtered and crystallized from ethanol (70%) to afford pure coumarin acetohydrazide derivative 4.

Yield: 78%; m.p.: 290–292 °C; ^1^H-NMR (400 MHz, DMSO-*d*_6_, ppm) *δ*: 10.30 (s, 1H, NH), 7.71 (d, *J* = 8.8 Hz, 1H, coumarin H-5), 7.01 (d, *J* = 8.9 Hz, 1H, coumarin H-6), 6.96 (s, 1H, coumarin H-3), 4.76 (s, 2H, OCH_2_), 2.36 (s, 3H, CH_3_), 2.07 (s, 3H, CH_3_). ^13^C-NMR (DMSO, 101 MHz, ppm) *δ*: 166.70, 161.61, 160.01, 153.23, 147.21, 126.64, 118.72, 114.66, 112.89, 101.85, 66.71, 15.40, 13.40. Analysis for C_13_H_14_N_2_O_4_ (262.26): calcd, C, 59.54; H, 5.38; N, 10.68; found, C, 59.58; H, 5.43; N, 10.57.

#### 4.1.3. General procedure for the synthesis of (*Z*)-*N*-(1-aryl)-(3-(2-(2-(4,8-dimethyl-2-oxo-2*H*-chromen-7-yloxy)acetyl)hydrazinyl)-3-oxoprop-1-en-2-yl)arylamides (5 and 6a–f)

To a suspension of the coumarin acetohydrazinyl derivative 4 (0.262 g, 1 mmol) in absolute ethanol (20 mL), an equimolar amount of the appropriate ethyl 2-(arylamido)-3-aryl acrylate derivative and few drops of glacial acetic acid (10 drops) were added. The reaction mixture was heated to reflux for 6–8 h. After completion of the reaction as indicated by TLC using a solvent mixture of hexane and ethyl acetate in a ratio of 1 : 4, the reaction mixture was left to cool to room temperature. The separated solid was filtered, washed with ether and crystallized from ethanol (70%) to afford pure coumarin-acrylamide derivatives 5 and 6a–f.

##### 4.1.3.1. (*Z*)-*N*-(3-(2-(2-(4,8-Dimethyl-2-oxo-2*H*-chromen-7-yloxy)acetyl)hydrazinyl)-1-(furan-2-yl)-3-oxoprop-1-en-2-yl)-3,4-dimethoxybenzamide (5)

Yield: 71%; m.p.: 219–221 °C; ^1^H-NMR (400 MHz, DMSO-*d*_6_, ppm) *δ*: 10.33 (d, *J* = 31.4 Hz, 1H, NH), 10.08 (d, *J* = 72.4 Hz, 1H, NH), 9.65 (d, *J* = 69.5 Hz, 1H, NH), 7.80 (d, *J* = 21.6 Hz, 1H, coumarin H-5), 7.73–7.60 (m, 3H, furan CH and Ar–H), 7.17 (s, 1H, olefinic CH), 7.11–7.05 (m, 1H, Ar–H), 7.01 (d, *J* = 8.8 Hz, 1H, coumarin H-6), 6.96 (s, 1H, coumarin H-3), 6.78 (dd, *J* = 37.2, 3.1 Hz, 1H, furan CH), 6.61 (d, *J* = 15.2 Hz, 1H, furan CH), 4.77 (d, *J* = 25.0 Hz, 2H, OCH_2_), 3.84 (s, 6H, 2OCH_3_), 2.36 (s, 3H, CH_3_), 2.07 (s, 3H, CH_3_). ^13^C-NMR (DMSO, 101 MHz, ppm) *δ*: 170.67, 166.20, 165.30, 163.89, 161.17, 159.69, 152.77, 151.66, 149.59, 148.17, 146.76, 144.69, 126.18, 126.13, 126.06, 121.44, 118.20, 118.10, 114.23, 114.13, 112.42, 111.33, 110.88, 101.35, 66.19, 55.67, 55.62, 14.92, 12.92. Analysis for C_29_H_27_N_3_O_9_ (561.54): calcd, C, 62.03; H, 4.85; N, 7.48; found, C, 61.88; H, 4.94; N, 7.56.

##### 4.1.3.2. (*Z*)-*N*-(3-(2-(2-(4,8-Dimethyl-2-oxo-2*H*-chromen-7-yloxy)acetyl)hydrazinyl)-1-(4-fluorophenyl)-3-oxoprop-1-en-2-yl)-3,4-dimethoxybenzamide (6a)

Yield: 66%; m.p.: 236–238 °C; ^1^H-NMR (400 MHz, DMSO-*d*_6_, ppm) *δ*: 10.21 (d, *J* = 44.6 Hz, 2H, 2NH), 9.73 (d, *J* = 109.2 Hz, 1H, NH), 7.72 (d, *J* = 9.0 Hz, 1H, coumarin H-5), 7.69–7.58 (m, 3H, Ar–H), 7.26 (d, *J* = 9.8 Hz, 2H, Ar–H), 7.22 (d, *J* = 8.8 Hz, 1H, Ar–H), 7.13–7.08 (m, 1H, olefinic CH), 7.08–6.99 (m, 2H, Ar–H and coumarin H-6), 6.97 (d, *J* = 2.2 Hz, 1H, coumarin H-3), 4.80 (d, *J* = 35.2 Hz, 2H, OCH_2_), 3.84 (s, 3H, OCH_3_), 3.83 (s, 3H, OCH_3_), 2.37 (s, 3H, CH_3_), 2.08 (s, 3H, CH_3_). ^13^C-NMR (DMSO, 101 MHz, ppm) *δ*: 166.65 (d, *J*_C-F_ = 138.2 Hz), 165.98, 164.93, 163.69, 161.62, 160.16, 153.24, 152.20, 148.64, 147.22, 132.15, 132.06 (d, *J*_C-F_ = 8.4 Hz), 131.09, 131.06, 129.25, 128.98 (d, *J*_C-F_ = 5.4 Hz), 126.60, 126.24, 121.92, 118.66, 116.13, 115.91 (d, *J*_C-F_ = 21.6 Hz), 114.59, 112.88, 111.79, 111.34, 66.65, 56.14, 56.07, 15.38, 13.39. Analysis for C_31_H_28_FN_3_O_8_ (589.57): calcd, C, 63.15; H, 4.79; N, 7.13; found, C, 63.22; H, 4.83; N, 7.03.

##### 4.1.3.3. (*Z*)-*N*-(3-(2-(2-(4,8-Dimethyl-2-oxo-2*H*-chromen-7-yloxy)acetyl)hydrazinyl)-1-(4-fluorophenyl)-3-oxoprop-1-en-2-yl)-3,4,5-trimethoxybenzamide (6b)

Yield: 74%; m.p.: 238–240 °C; ^1^H-NMR (400 MHz, DMSO-*d*_6_, ppm) *δ*: 10.29 (s, 2H, 2NH), 8.38 (dd, *J* = 8.6, 5.8 Hz, 2H, Ar–H), 7.72 (d, *J* = 8.9 Hz, 1H, coumarin H-5), 7.40 (d, *J* = 8.9 Hz, 1H, Ar–H), 7.37 (s, 1H, olefinic CH), 7.36 (d, *J* = 5.0 Hz, 1H, Ar–H), 7.26 (d, *J* = 10.3 Hz, 1H, coumarin H-6), 7.02 (dd, *J* = 8.8, 2.5 Hz, 2H, Ar–H), 6.97 (s, *J* = 2.4 Hz, 1H, coumarin H-3), 4.76 (s, 2H, OCH_2_), 3.92 (s, 6H, 2OCH_3_), 3.81 (s, 3H, OCH_3_), 2.37 (s, 3H, CH_3_), 2.08 (s, 3H, CH_3_). ^13^C-NMR (DMSO, 101 MHz, ppm) *δ*: 167.34 (d, *J*_C–F_ = 164.8 Hz), 166.70, 165.20, 163.26, 162.70, 161.60, 160.00, 153.75, 153.23, 147.20, 142.86, 135.31 (d, *J*_C–F_ = 8.9 Hz), 133.17, 130.59 (d, *J*_C–F_ = 27.2 Hz), 129.57, 126.63, 120.44, 118.72, 116.79 (d, *J*_C–F_ = 21.9 Hz), 116.57, 114.65, 112.88, 105.83, 101.85, 66.70, 60.84, 56.71, 15.39, 13.39. Analysis for C_32_H_30_FN_3_O_9_ (619.59): calcd, C, 62.03; H, 4.88; N, 6.78; found, C, 62.11; H, 5.02; N, 6.64.

##### 4.1.3.4. (*Z*)-*N*-(1-(4-Chlorophenyl)-3-(2-(2-(4,8-dimethyl-2-oxo-2*H*-chromen-7-yloxy)acetyl)hydrazinyl)-3-oxoprop-1-en-2-yl)-3,4,5-trimethoxybenzamide (6c)

Yield: 68%; m.p.: 247–249 °C; ^1^H-NMR (400 MHz, DMSO-*d*_6_, ppm) *δ*: 10.40 (d, *J* = 15.6 Hz, 1H, NH), 10.26 (s, 1H, NH), 9.79 (d, *J* = 14.4 Hz, 1H, NH), 7.73 (d, *J* = 8.3 Hz, 1H, coumarin H-5), 7.68 (d, *J* = 8.1 Hz, 2H, Ar–H), 7.48 (d, *J* = 8.1 Hz, 2H, Ar–H), 7.38 (d, *J* = 7.0 Hz, 1H, olefinic CH), 7.35 (s, 2H, Ar–H), 7.02 (d, *J* = 8.3 Hz, 1H, coumarin H-6), 6.98 (s, 1H, coumarin H-3), 4.80 (s, 2H, OCH_2_), 3.85 (s, 6H, 2OCH_3_), 3.74 (s, 3H, OCH_3_), 2.37 (s, 3H, CH_3_), 2.08 (s, 3H, CH_3_). ^13^C-NMR (DMSO, 101 MHz, ppm) *δ*: 166.69, 165.81, 164.74, 161.63, 160.16, 153.24, 153.02, 147.23, 140.93, 134.26, 133.76, 133.37, 131.58, 129.97, 129.13, 128.68, 126.62, 118.68, 114.60, 112.89, 106.09, 101.82, 66.64, 60.57, 56.54, 15.40, 13.40. Analysis for C_32_H_30_ClN_3_O_9_ (636.05): calcd, C, 60.43; H, 4.75; N, 6.61; found, C, 60.29; H, 4.58; N, 6.69.

##### 4.1.3.5. (*Z*)-*N*-(1-(4-Cyanophenyl)-3-(2-(2-(4,8-dimethyl-2-oxo-2*H*-chromen-7-yloxy)acetyl)hydrazinyl)-3-oxoprop-1-en-2-yl)-3,4,5-trimethoxybenzamide (6d)

Yield: 63%; m.p.: 246–248 °C; ^1^H-NMR (400 MHz, DMSO-*d*_6_, ppm) *δ*: 10.36 (s, 2H, 2NH), 9.86 (d, *J* = 175.5 Hz, 1H, NH), 7.86 (dt, *J* = 14.6, 7.4 Hz, 2H, Ar–H and coumarin H-5), 7.77 (t, *J* = 13.2 Hz, 2H, Ar–H), 7.71 (d, *J* = 8.9 Hz, 1H, Ar–H), 7.34 (d, *J* = 10.6 Hz, 2H, Ar–H), 7.22 (s, 1H, olefinic CH), 7.01 (d, *J* = 6.9 Hz, 1H, coumarin H-6), 6.97 (s, 1H, coumarin H-3), 4.81 (d, *J* = 37.5 Hz, 2H, OCH_2_), 3.84 (s, 6H, 2OCH_3_), 3.73 (s, 3H, OCH_3_), 2.36 (s, 3H, CH_3_), 2.07 (s, 3H, CH_3_). ^13^C-NMR (DMSO, 101 MHz, ppm) *δ*: 166.23, 165.37, 164.06, 161.15, 159.68, 152.77, 152.57, 146.74, 140.58, 138.89, 132.43, 131.63, 129.96, 128.33, 127.13, 126.13, 118.66, 118.21, 114.13, 112.42, 110.71, 105.66, 101.34, 66.16, 60.11, 56.08, 14.92, 12.92. Analysis for C_33_H_30_N_4_O_9_ (626.61): calcd, C, 63.25; H, 4.83; N, 8.94; found, C, 63.33; H, 4.95; N, 8.83.

##### 4.1.3.6. (*Z*)-*N*-(3-(2-(2-(4,8-Dimethyl-2-oxo-2*H*-chromen-7-yloxy)acetyl)hydrazinyl)-1-(4-methoxyphenyl)-3-oxoprop-1-en-2-yl)-3,4,5-trimethoxybenzamide (6e)

Yield: 69%; m.p.: 232–234 °C; ^1^H-NMR (400 MHz, DMSO-*d*_6_, ppm) *δ*: 10.30 (s, 2H, 2NH), 9.87 (d, *J* = 17.5 Hz, 1H, NH), 7.69 (d, *J* = 1.9 Hz, 1H, coumarin H-5), 7.63–7.55 (m, 2H, Ar–H), 7.38 (s, 2H, olefinic CH), 7.28 (d, *J* = 11.7 Hz, 1H, Ar–H), 7.01 (d, *J* = 6.6 Hz, 2H, coumarin H-6 and H-3), 6.97 (s, 2H, Ar–H), 4.76 (s, 2H, OCH_2_), 3.86 (s, 6H, 2OCH_3_), 3.76 (s, 3H, OCH_3_), 3.74 (s, 3H, OCH_3_), 2.36 (s, 3H, CH_3_), 2.08 (s, 3H, CH_3_). ^13^C-NMR (DMSO, 101 MHz, ppm) *δ*: 166.67, 165.78, 165.00, 161.61, 160.26, 160.01, 153.23, 152.98, 147.21, 131.76, 130.57, 130.38, 129.41, 127.22, 126.95, 126.63, 118.71, 114.65, 114.58, 112.89, 106.05, 101.84, 66.72, 60.57, 56.52, 55.69, 15.39, 13.40. Analysis for C_33_H_33_N_3_O_10_ (631.63): calcd, C, 62.75; H, 5.27; N, 6.65; found, C, 62.55; H, 5.16; N, 6.77.

##### 4.1.3.7. (*Z*)-*N*-(1-(3,4-Dimethoxyphenyl)-3-(2-(2-(4,8-dimethyl-2-oxo-2*H*-chromen-7-yloxy)acetyl)hydrazinyl)-3-oxoprop-1-en-2-yl)-3,4,5-trimethoxybenzamide (6f)

Yield: 59%; m.p.: 218–220 °C; ^1^H-NMR (400 MHz, DMSO-*d*_6_, ppm) *δ*: 10.34 (d, *J* = 41.6 Hz, 1H, NH), 10.16 (d, *J* = 20.4 Hz, 1H, NH), 9.72 (d, *J* = 97.9 Hz, 1H, NH), 7.75 (s, 2H, Ar–H), 7.69 (d, *J* = 1.9 Hz, 1H, coumarin H-5), 7.57 (d, *J* = 1.8 Hz, 1H, Ar–H), 7.20 (s, 1H, olefinic CH), 7.07 (d, *J* = 8.5 Hz, 1H, Ar–H), 7.04 (d, *J* = 3.5 Hz, 1H, coumarin H-6), 6.98 (d, *J* = 4.8 Hz, 2H, coumarin H-3 and Ar–H), 4.78 (d, *J* = 25.6 Hz, 2H, OCH_2_), 3.88 (s, 6H, 2OCH_3_), 3.86 (s, 3H, OCH_3_), 3.77 (s, 3H, OCH_3_), 3.62 (s, 3H, OCH_3_), 2.37 (s, 3H, CH_3_), 2.08 (s, 3H, CH_3_). ^13^C-NMR (DMSO, 101 MHz, ppm) *δ*: 167.03, 166.13, 162.34, 159.70, 153.48, 152.78, 151.67, 148.97, 148.09, 140.11, 132.14, 129.41, 128.97, 127.99, 126.13, 122.14, 118.19, 116.98, 114.12, 112.44, 111.90, 109.60, 107.17, 101.34, 66.23, 60.24, 60.04, 55.73, 55.59, 14.92, 12.92. Analysis for C_34_H_35_N_3_O_11_ (661.66): calcd, C, 61.72; H, 5.33; N, 6.35; found, C, 61.87; H, 5.52; N, 6.18.

### 4.2. Biological studies

#### 4.2.1. *In vitro* cytotoxicity assay

The target coumarin-acrylamide hybrids 5 and 6a–f reported in this work were evaluated for their potential cytotoxic activity (ESI†).

#### 4.2.2. Tubulin assay

The active coumarin-acrylamide hybrids 6b, 6e, and 6f were evaluated for their anti-tubulin activity utilizing podophyllotoxin as reference standard (ESI†).

#### 4.2.3. Cell cycle study

The most active coumarin-acrylamide hybrid 6e was assessed in a cell cycle study with HepG2 cancerous liver cells in accordance with the manufacturer's instructions (ESI†).

#### 4.2.4. Cell apoptosis study

Cell apoptosis assay of the most active coumarin-acrylamide hybrid 6e against HepG2 cells was carried out in accordance with the manufacturer's instructions (ESI†).

#### 4.2.5. *In vivo* histopathological studies

See ESI.†

### 4.3. Molecular docking study

See ESI.†

## Conflicts of interest

The authors report no conflicts of interest.

## Supplementary Material

RA-013-D3RA06644D-s001
