# Supplementary material for: Design, synthesis and antiproliferative screening of newly synthesized coumarin-acrylamide hybrids as potential cytotoxic and apoptosis inducing agents
Source: RSC Adv. 2023 Nov 6;13(46):32547–57. doi: 10.1039/d3ra06644d (PMC10626558; doi:10.1039/d3ra06644d)
Supplement: RA-013-D3RA06644D-s001 [file RA-013-D3RA06644D-s001.pdf]

Electronic Supplementary Material (ESI) for RSC Advances.
This journal is © The Royal Society of Chemistry 2023

Supporting Information

Design, Synthesis and Antiproliferative Screening of Newly Synthesized Coumarin-Acrylamide Hybrids as Potential Cytotoxic and Apoptosis Inducing Agents

Hany M. Abd El-Lateef [1,2,*], Lina M. A. Abdel Ghany [3], Rasha Mohammed Saleem [4], Ali Hassan Ahmed Maghrabi [5], Maryam Abdulrahman Yahya Alahdal [6], Eman Hussain Khalifa Ali [7], Botros Y. Beshay [8], Islam Zaki [9,*] and Reham E. Masoud [10]

[1] Department of Chemistry, College of Science, King Faisal University, Al-Ahsa 31982, Saudi Arabia; [2] Department of Chemistry, Faculty of Science, Sohag University, Sohag 82524, Egypt; [3] Pharmaceutical Chemistry Department, College of Pharmaceutical Sciences and Drug Manufacturing, Misr University for Science and Technology, 6th of October City, Egypt; [4] Department of Laboratory Medicine, Faculty of Applied Medical Sciences, Al-Baha University, Al-Baha- 65431, Saudi Arabia; [5] Department of Biology, Faculty of Applied Science, Umm Al-Qura University, Makkah 24381, Saudi Arabia; [6] Department of Biology, Faculty of Applied Science, Umm Al-Qura University, Makkah 24381, Saudi Arabia; [7] Department of Laboratory Medicine, Faculty of Applied Medical Sciences, Albaha University, Saudi Arabia; [8] Pharmaceutical Sciences (Pharmaceutical Chemistry) Department, College of Pharmacy, Arab Academy for Science, Technology and Maritime Transport, Alexandria, Egypt; [9] Pharmaceutical Organic Chemistry Department, Faculty of pharmacy, Port Said University, Port Said, Egypt; Clinical Pharmacology Department, Faculty of Medicine, Port Said University, Port Said 42526, Egypt.

** To whome correspondence should be addressed*

Islam Zaki, PhD. Pharmaceutical Organic Chemistry Department, Faculty of pharmacy, Port Said University, Port Said, Egypt.

***E-mail address*: Eslam.Zaki@pharm.psu.edu.eg (I. Zaki)**

Hany M. Abd El-Lateef, PhD. Department of Chemistry, College of Science, King Faisal University, Al-Ahsa 31982, Saudi Arabia.

***E-mail address*:** hmahmed@kfu.edu.sa

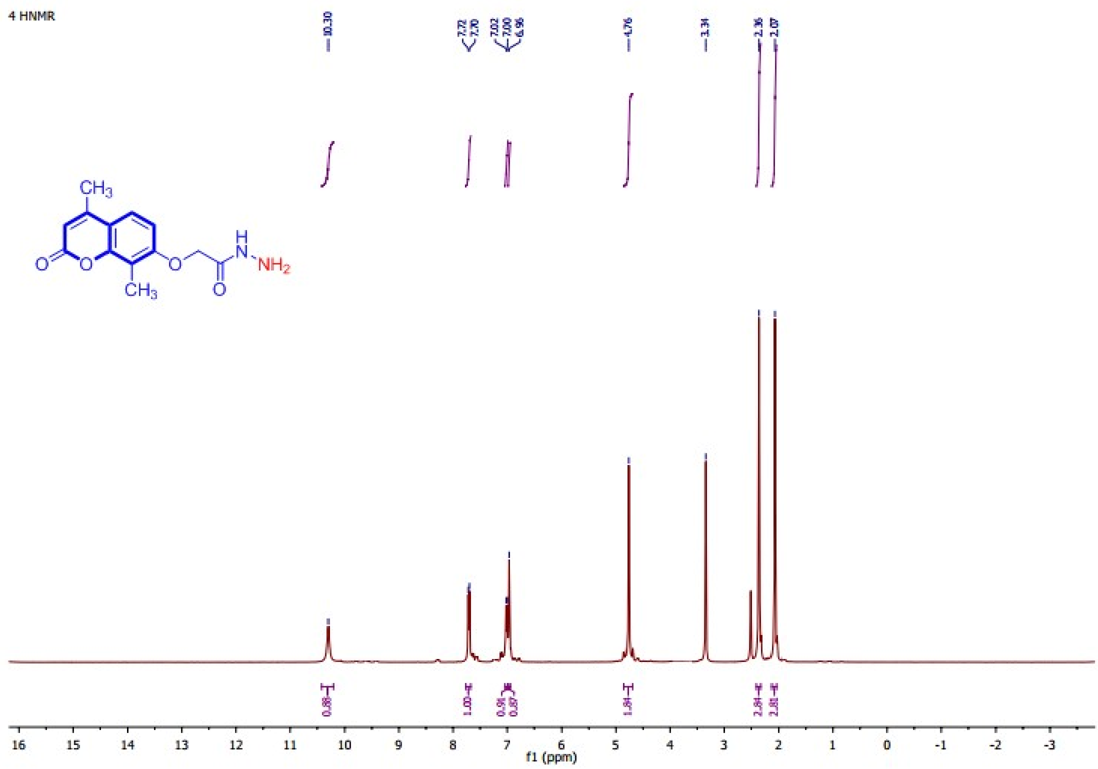

Figure S1: ^{1}H-NMR spectrum of compound **4** at 400 MHz in DMSO-d_6

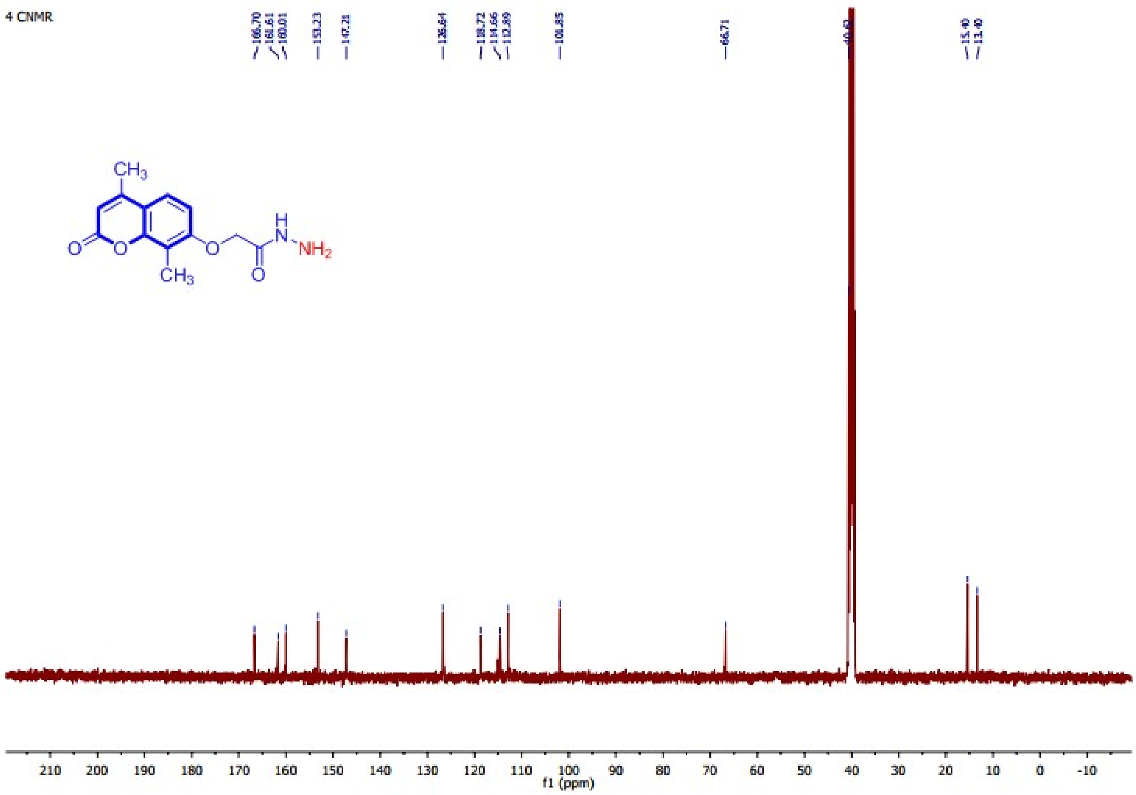

Figure S2: ^{13}C-NMR spectrum of compound **4** at 100 MHz in DMSO-d_6

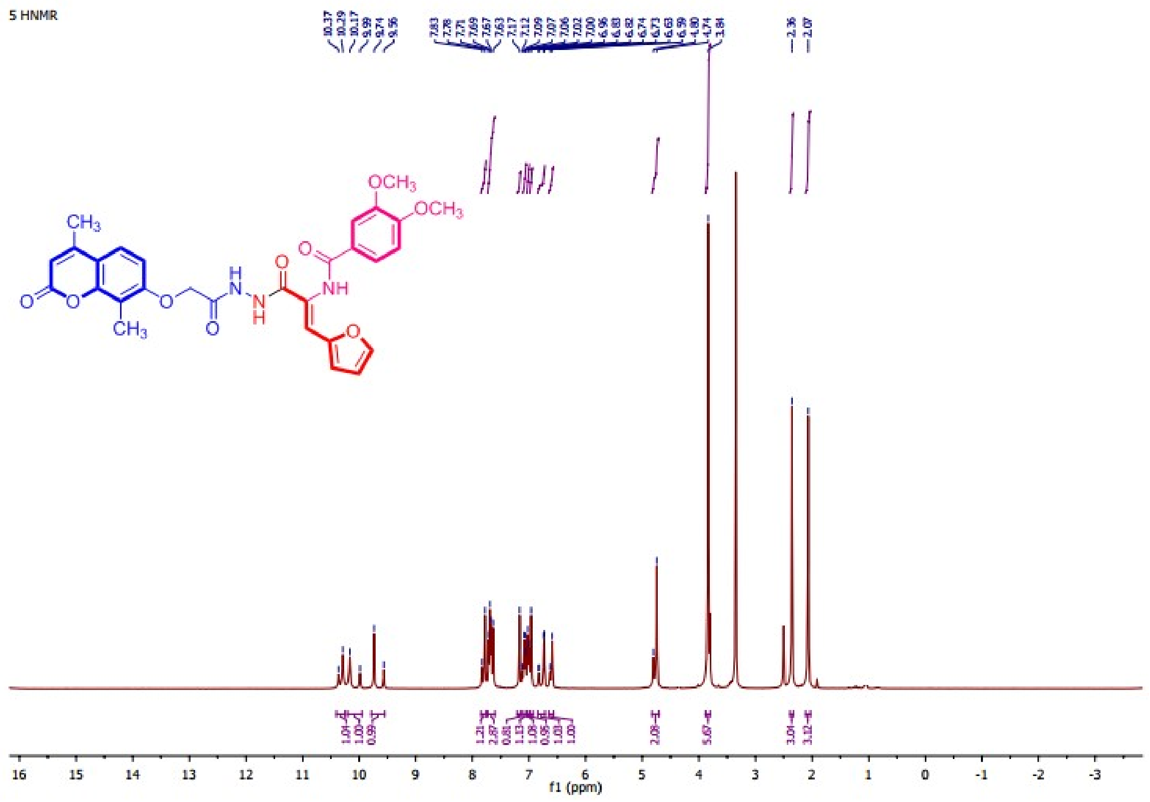

Figure S3: ^{1}H-NMR spectrum of compound **5** at 400 MHz in DMSO-d_6

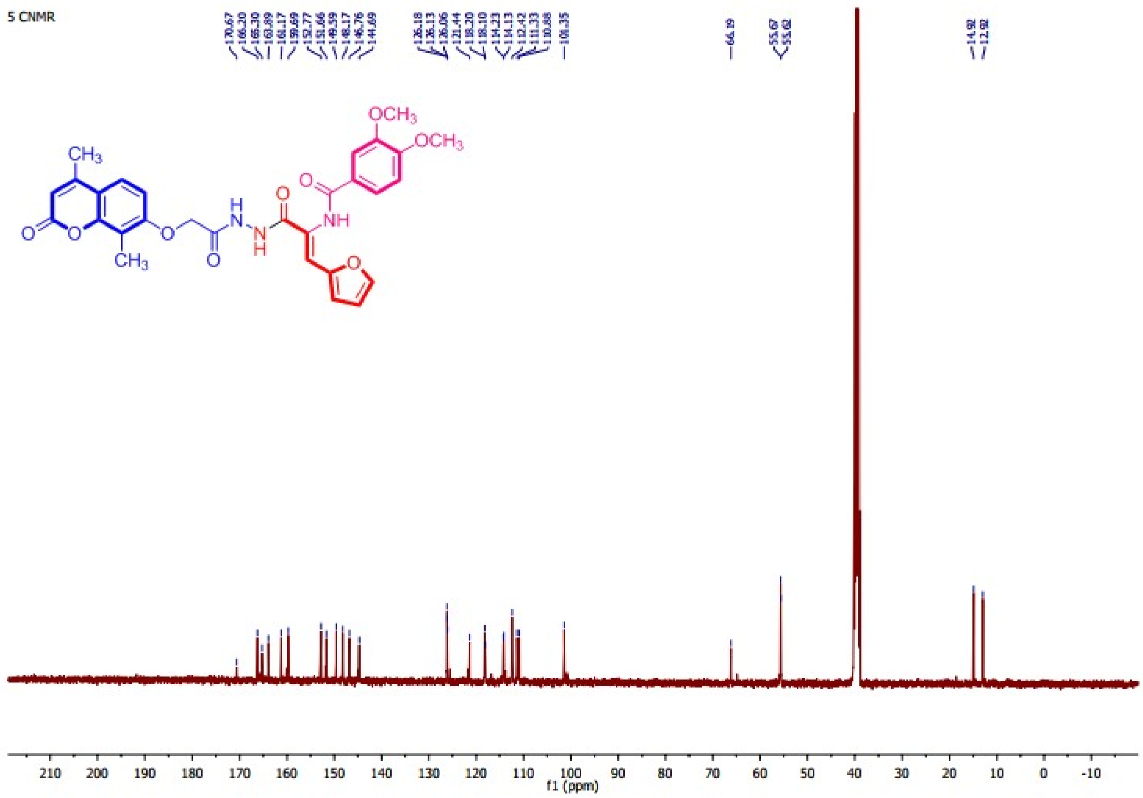

Figure S4: ^{13}C-NMR spectrum of compound **5** at 100 MHz in DMSO-d_6

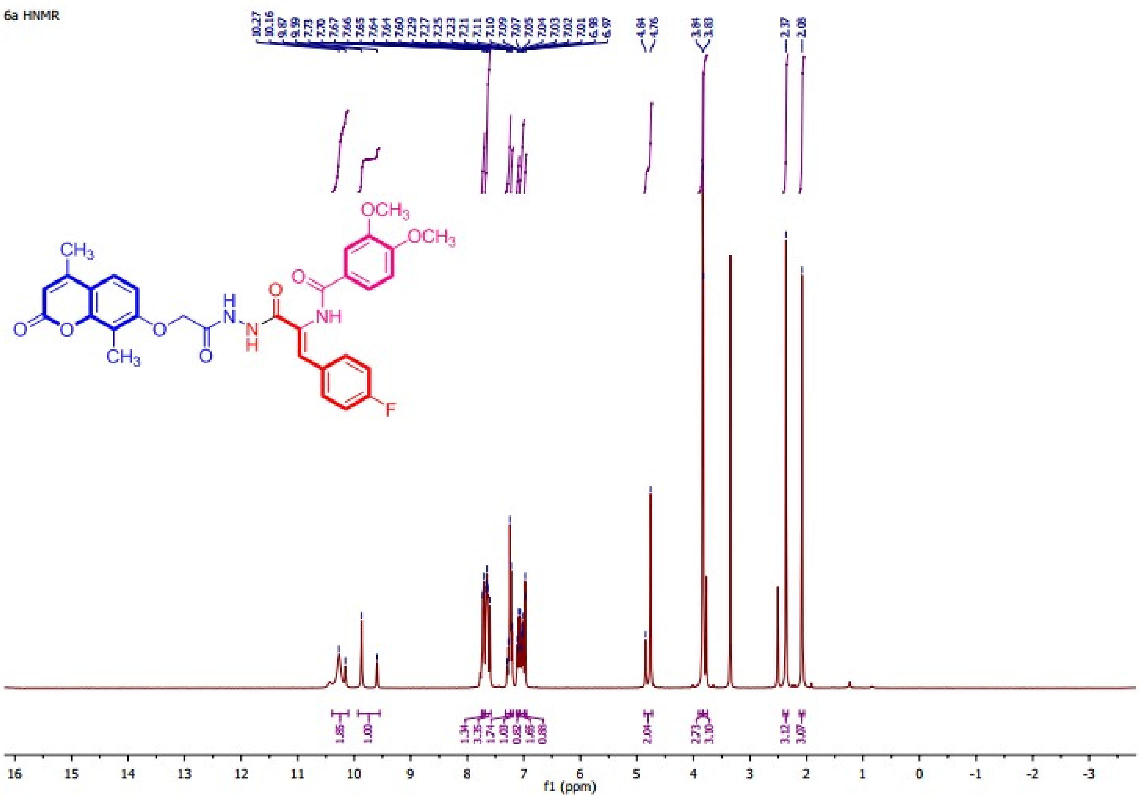

Figure S5: ^{1}H-NMR spectrum of compound **6a** at 400 MHz in DMSO-d_6

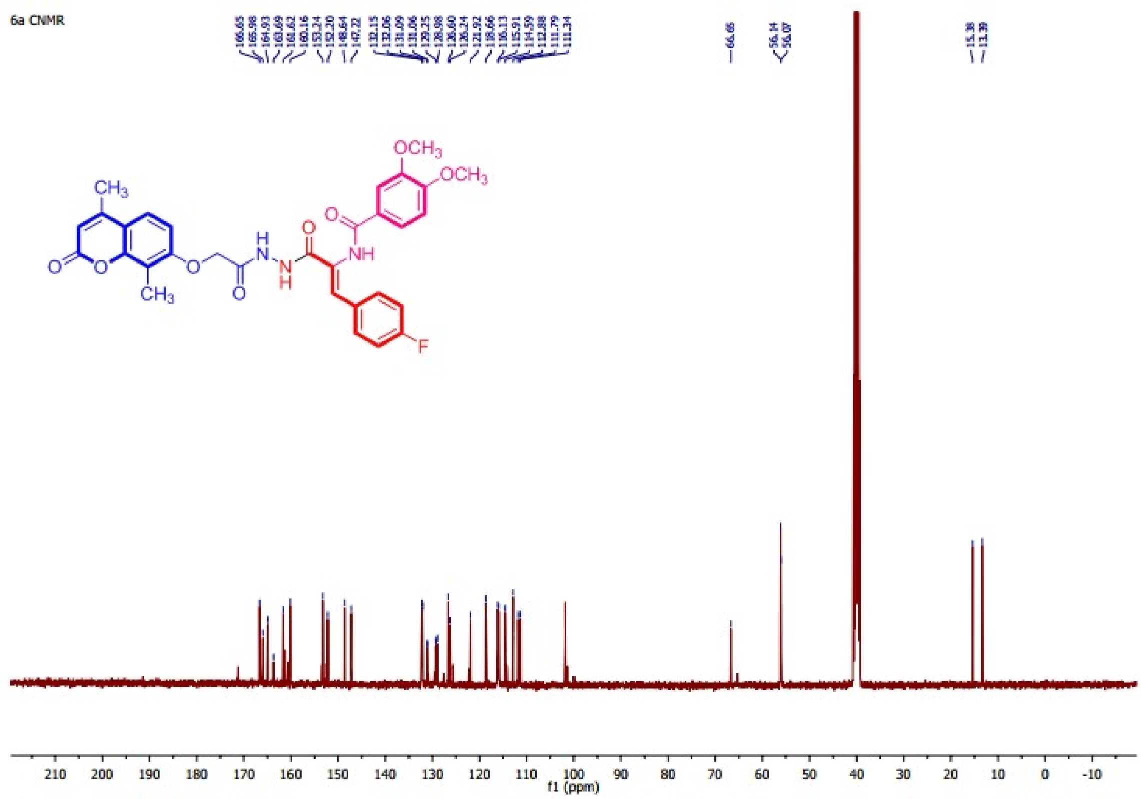

Figure S6: ^{13}C-NMR spectrum of compound **6a** at 100 MHz in DMSO-d_6

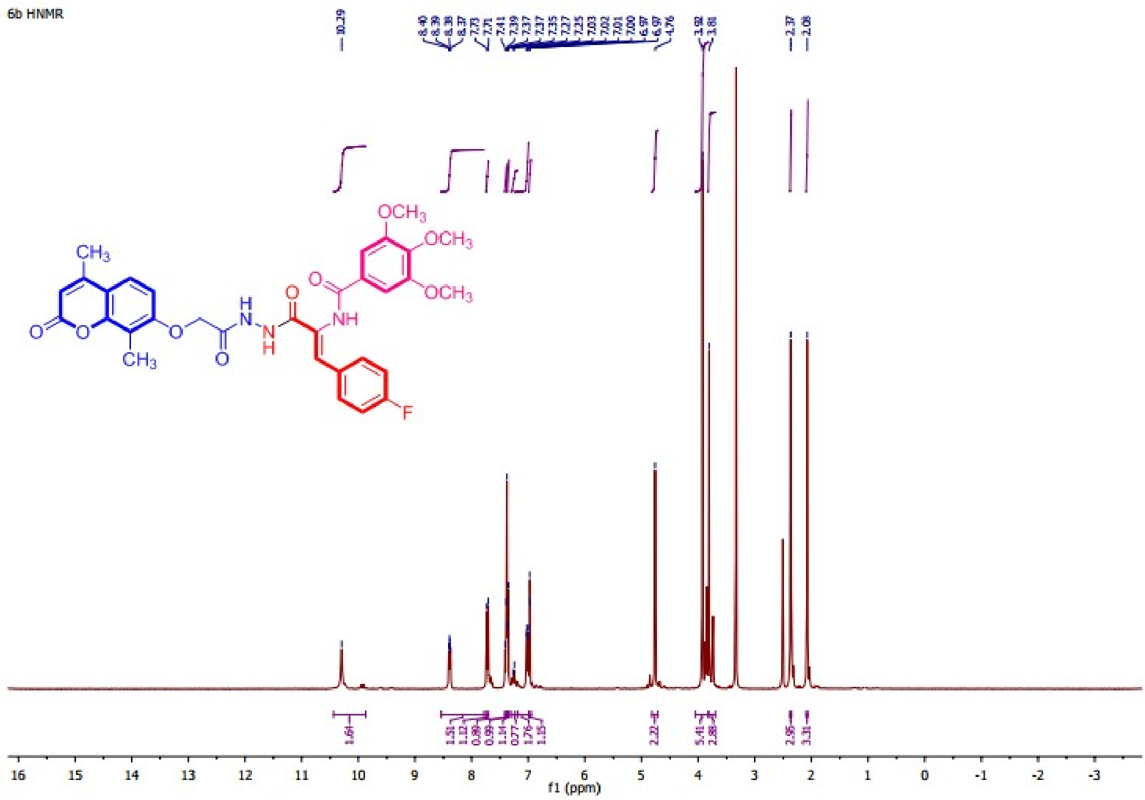

Figure S7: ^{1}H-NMR spectrum of compound **6b** at 400 MHz in DMSO-d_6

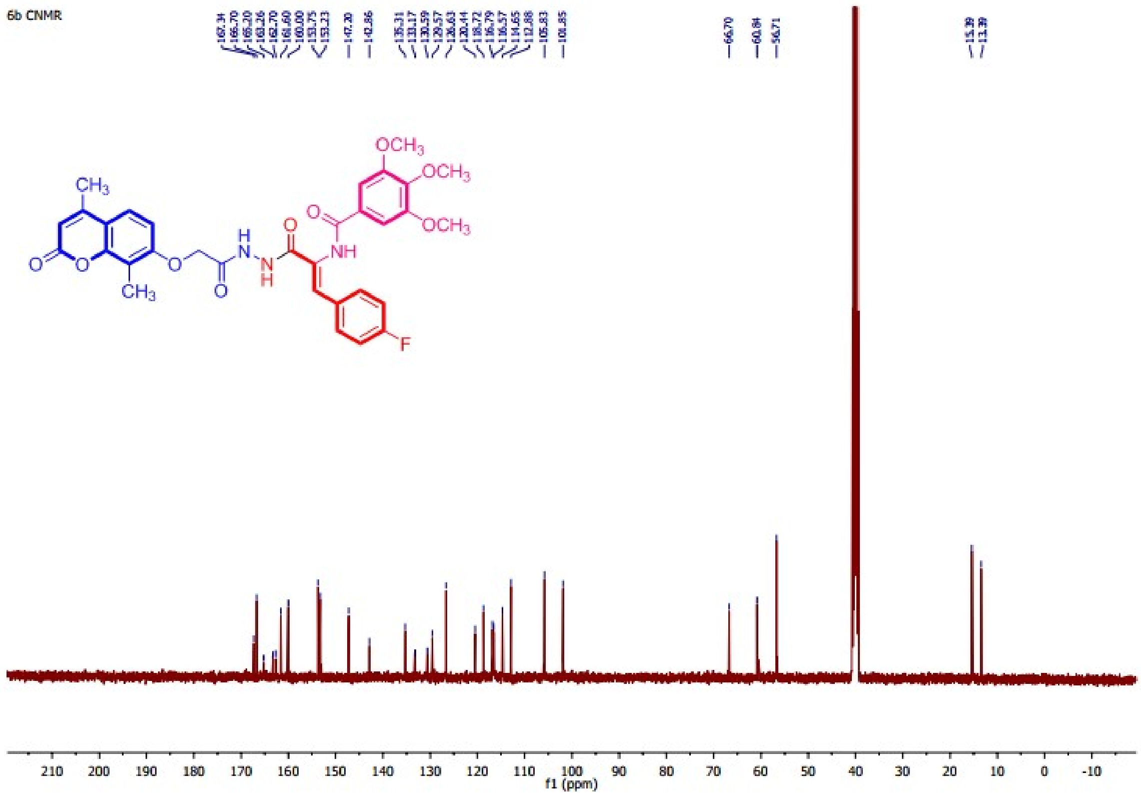

Figure S8: ^{13}C-NMR spectrum of compound **6b** at 100 MHz in DMSO-d_6

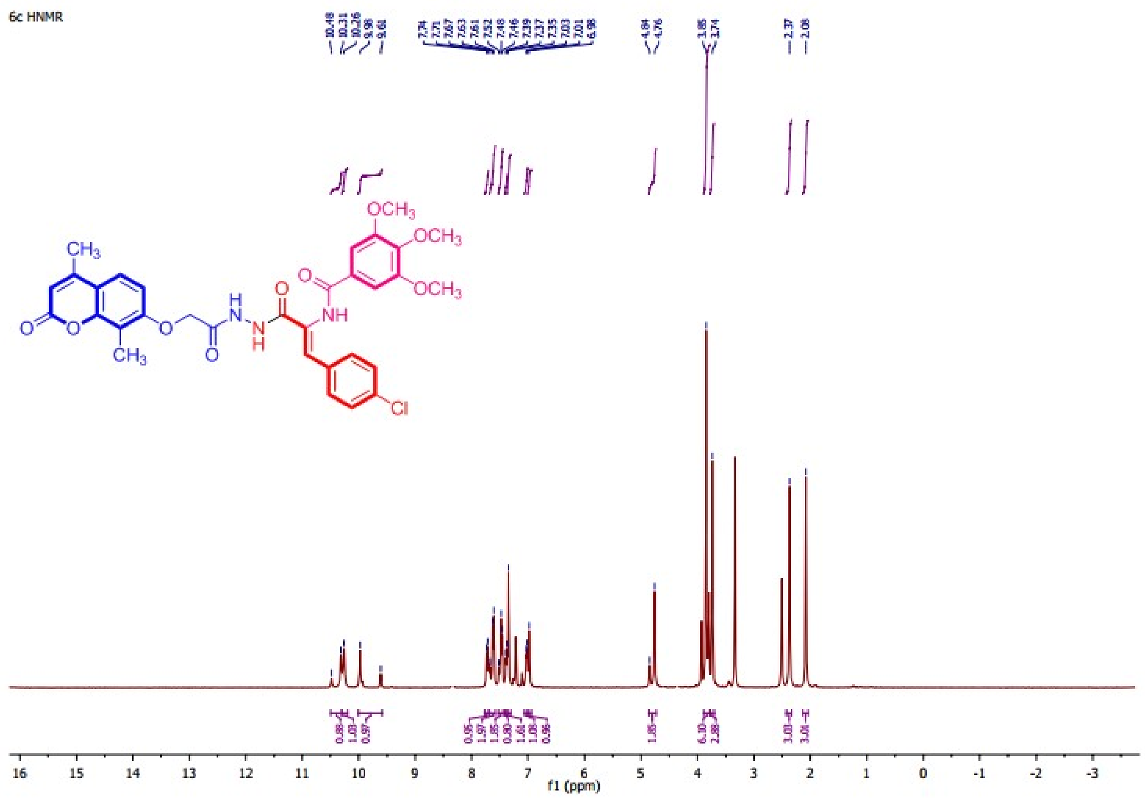

Figure S9: ^{1}H-NMR spectrum of compound **6c** at 400 MHz in DMSO-d_6

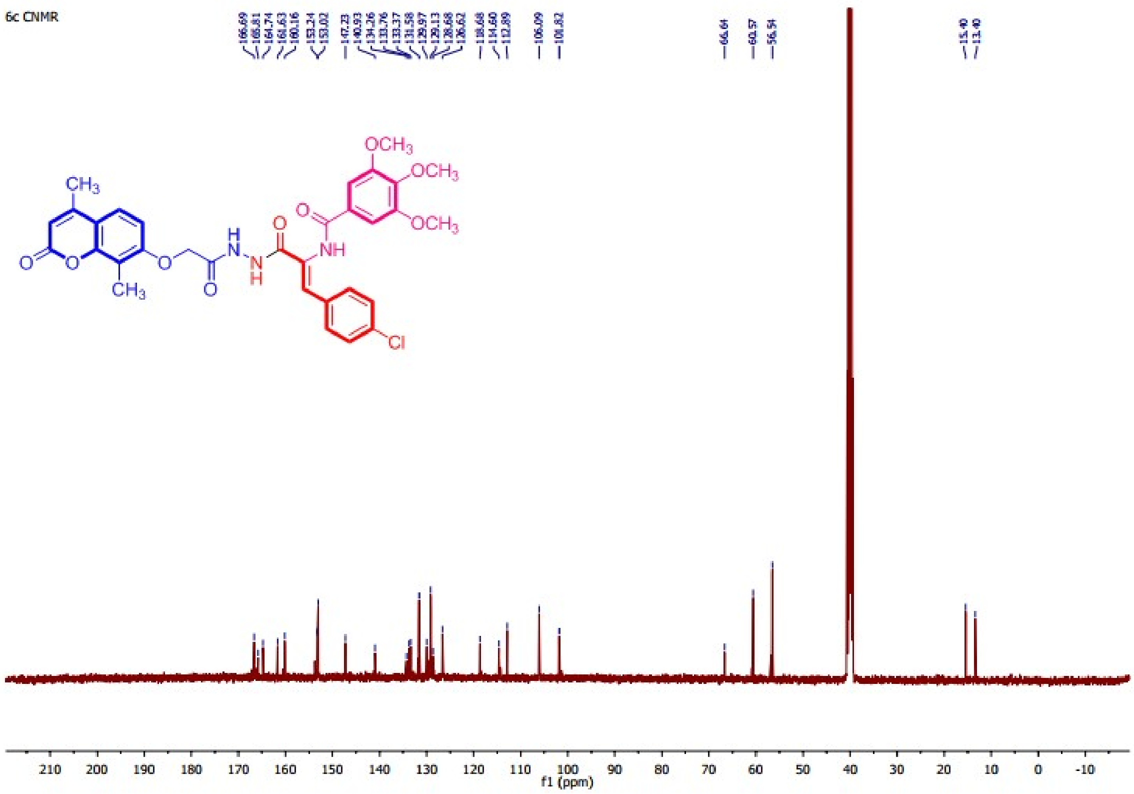

Figure S10: ^{13}C-NMR spectrum of compound **6c** at 100 MHz in DMSO-d_6

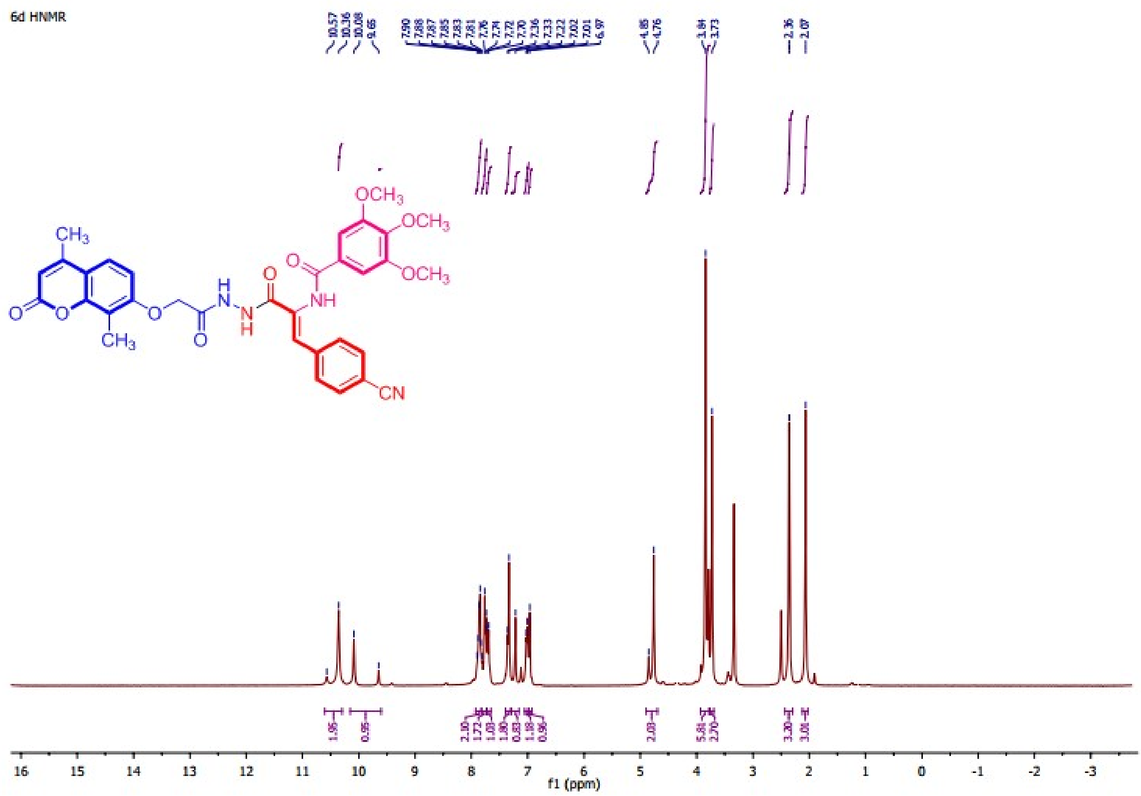

Figure S11: ^{1}H-NMR spectrum of compound **6d** at 400 MHz in DMSO-d_6

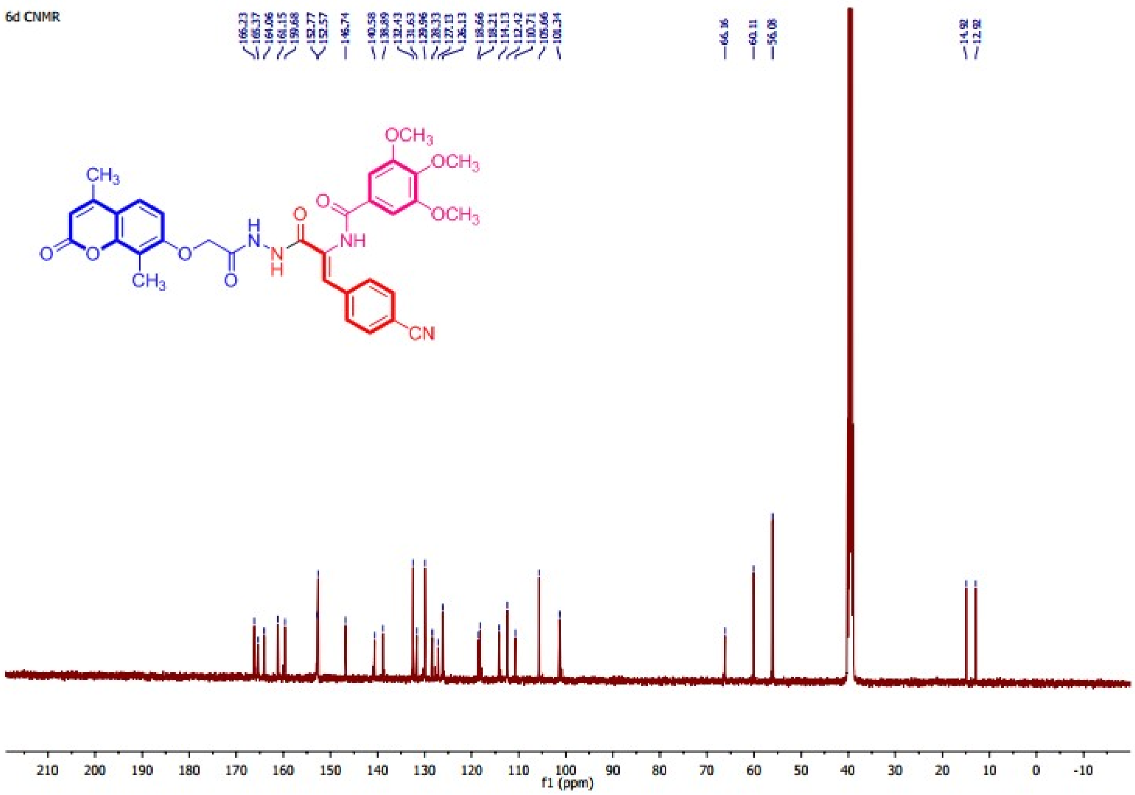

Figure S12: ^{13}C-NMR spectrum of compound **6d** at 100 MHz in DMSO-d_6

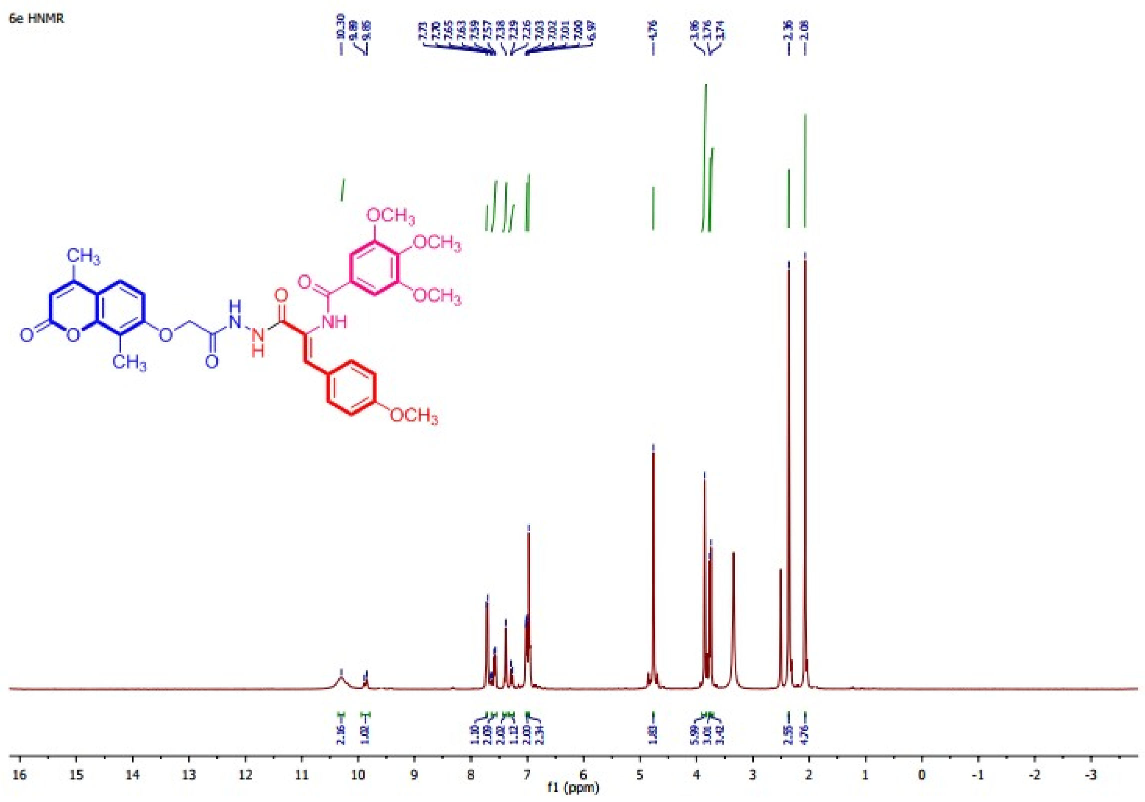

Figure S13: ^{1}H-NMR spectrum of compound **6e** at 400 MHz in DMSO-d_6

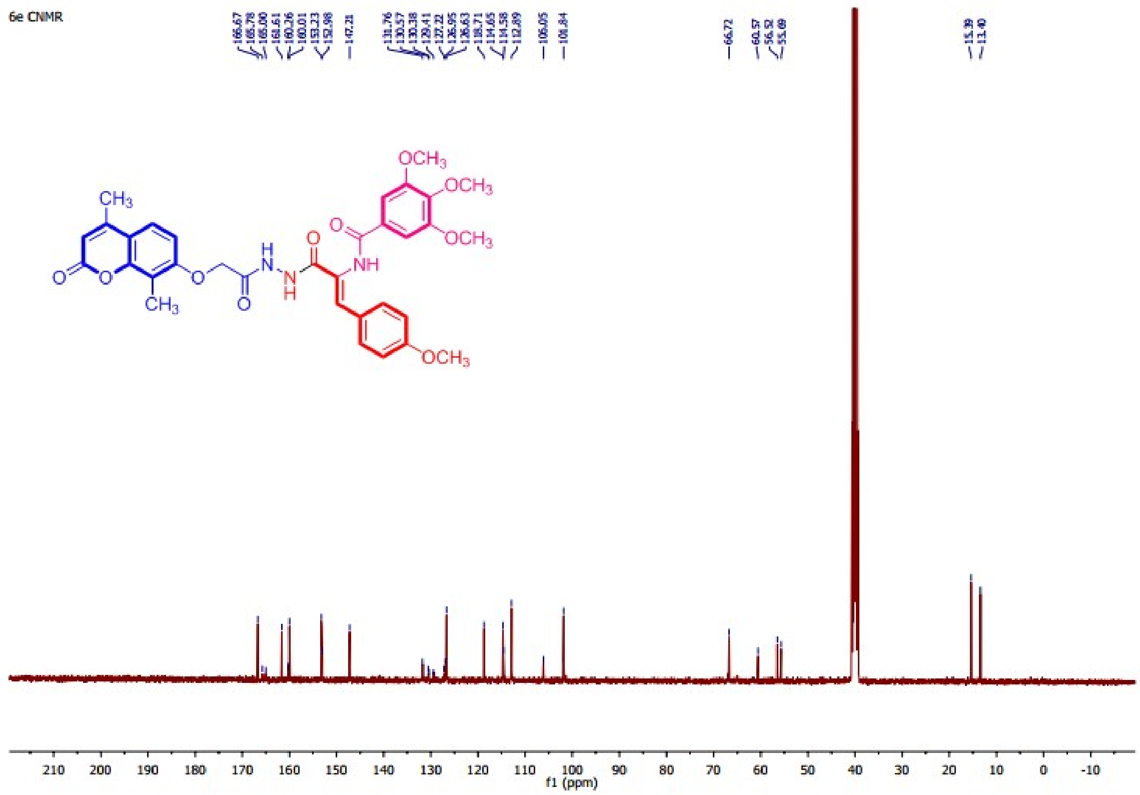

Figure S14: ^{13}C-NMR spectrum of compound **6e** at 100 MHz in DMSO-d_6

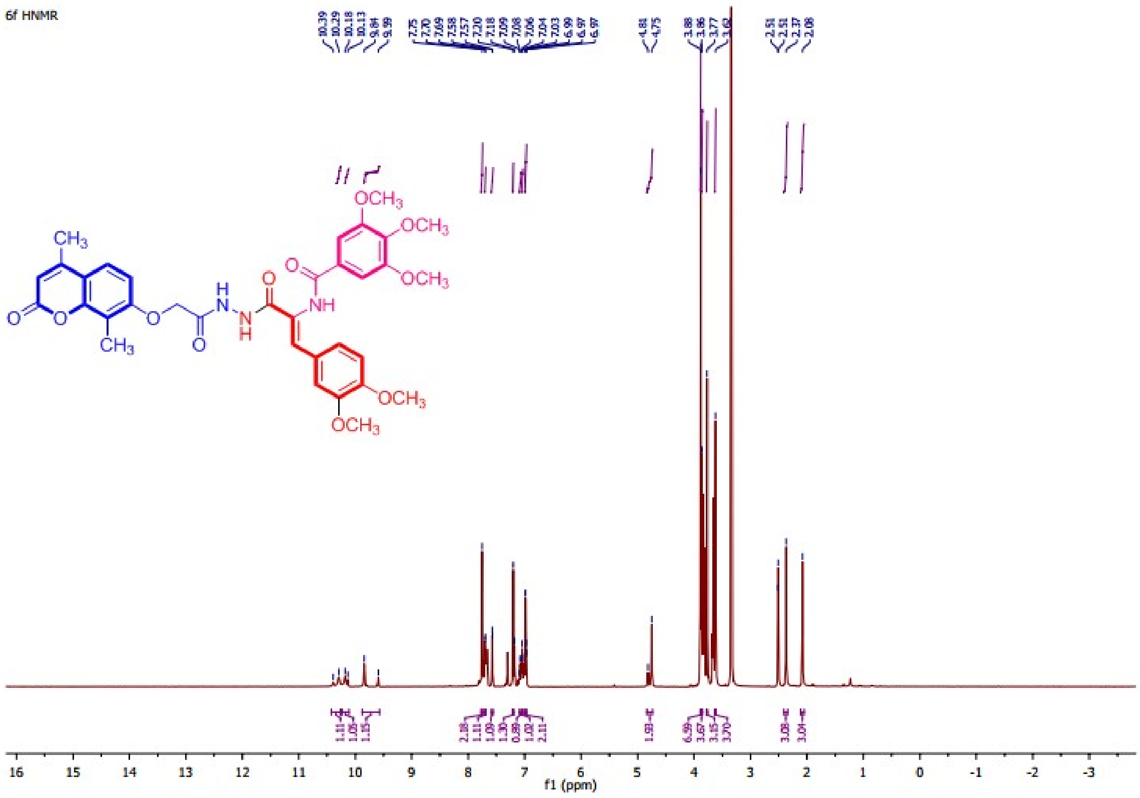

Figure S15: ^{1}H-NMR spectrum of compound **6f** at 400 MHz in DMSO-d_6

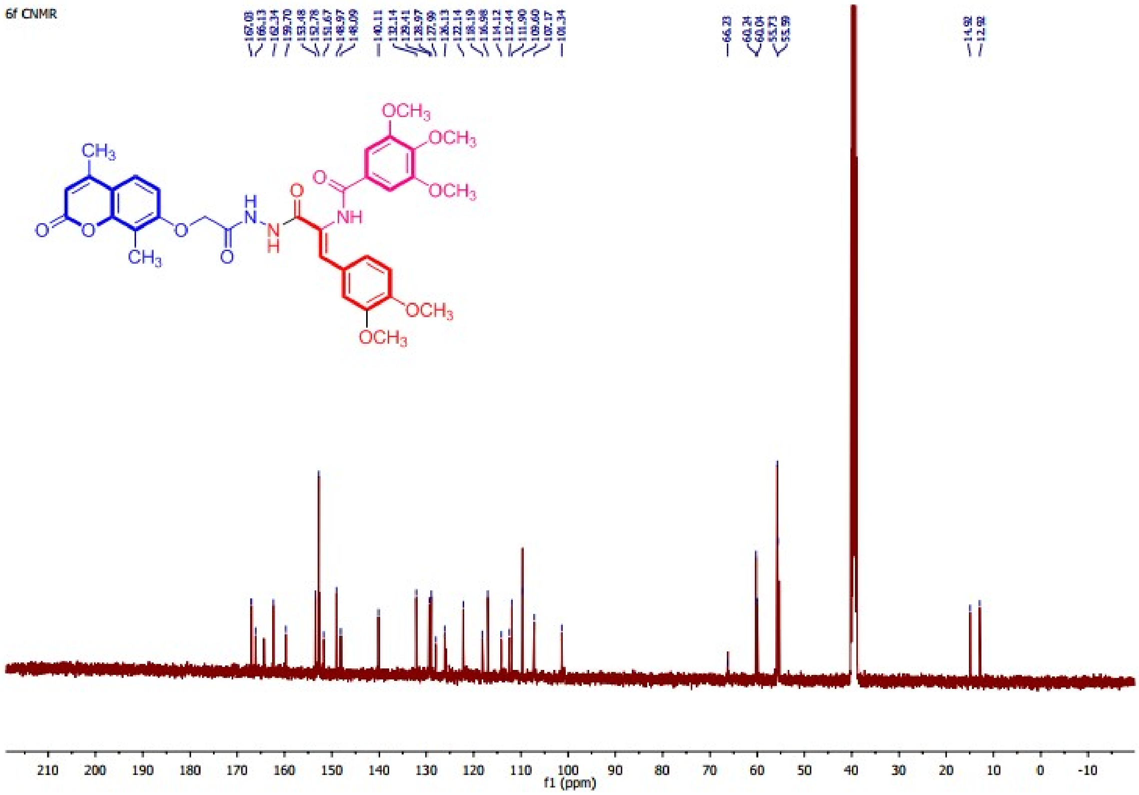

Figure S16: ^{13}C-NMR spectrum of compound **6f** at 100 MHz in DMSO-d_6

CTRL HepG2

Compd 6e/ HepG2

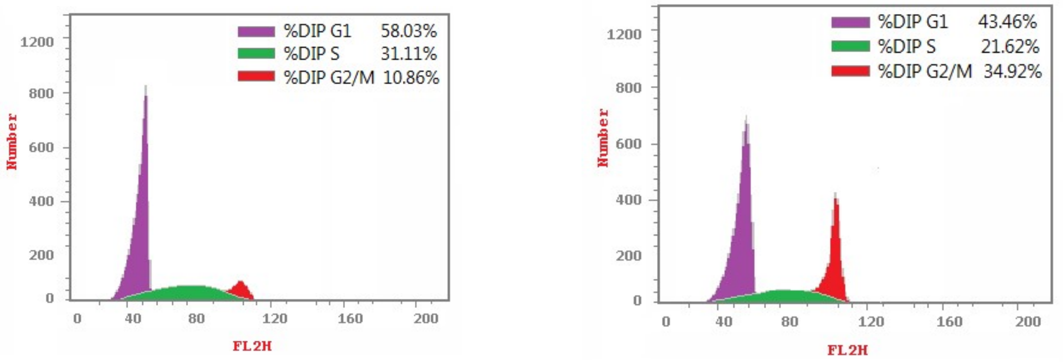

Figure S17: Cell cycle analysis of compound **6e** compared to untreated HepG2 cells.

Appendix A

S4.1. Chemistry

S4.1.1. General:

^{1}H-NMR and ^{13}C-NMR spectra were recorded with Brucker APX400 spectrometer in the ANARC research center faculty of Science, Zagazig University at 400 MHz and 100 MHz, respectively utilizing DMSO-d_6 as internal solvent. Chemical shifts were given on the δ scale and J values were reported in Hz. The elemental analyses were performed at the microanalytical unit at Faculty of Science, Cairo University. Chemical reagents and solvents were obtained from commercial source and used without further purification.

S4.2. Biological Studies

S4.2.1. Cytotoxic activity evaluation

To measure the cytotoxic actvity of the synthesized coumarin-acrylamide hybrids **5** and **6a-f** in liver cancer (HepG2) and normal liver (HL-7702) cell lines. Cell viability assay was assessed using MTT assay method. Cells at density of 1 x 10^4 were seeded in a 96-well plate at 37 ^{o}C for 24 h under 5% CO_2. After incubation, the cells were treated with different concentrations of the test coumarin-acrylamide derivatives **5** and **6a-f** and incubated for 24 h, then 20 µl of MTT solution at 5 mg/mL was applied and incubated for 4 h at 37 °C. Dimethyl sulphoxide (DMSO) in volume of 100 µl was added to each well to dissolve the purple formazan that had formed. The color intensity of the formazan product, which represents the growth condition of the cells, is quantified by using an ELISA plate reader (EXL 800, USA) at 570 nm absorbance. The experimental conditions were carried out with at least three replicates, and the experiments were repeated at least three times.

S4.2.2. Tubulin polymerization Assay

Compounds **6b, 6e** and **6f** and podophyllotoxin were evaluated for their tubulin inhibitory activity according to manufacturer's instructions using # abcam Human Beta-tubulin simplestep ELISA Kit ab245722.

S4.2.3. Cell cycle analysis of compound 6e

Cell cycle analysis in HepG2 cells was investigated using fluorescent Annexin V-FITC/ PI detection kit (*BioVision* EZCell™ Cell Cycle Analysis Kit Catalog #K920) by flow cytometry assay. HepG2 cells at a density of 2 × 10^5 per well were harvested and washed twice in PBS. After that, the cells were incubated at 37 °C and 5% CO_2. The medium was incubated with the tested compound **6e** at the IC_{50} (μM) for 48 h, washed twice in PBS, fixed with 70% ethanol, rinsed again with PBS. Afterward, medium was stained with DNA fluorochrome PI for 15 min at 37 °C. The samples were immediately analyzed using Facs Calibur flow cytometer (Becton and Dickinson, Heidelberg, Germany).

S4.2.4. Apoptosis assay for compound 6e

Apoptosis in HepG2 cells was investigated using fluorescent Annexin V-FITC/ PI detection kit (*BioVision* Annexin V-FITC Apoptosis Detection Kit, Catalog #: K101) by flow cytometry assay. HepG2 cells at a density of 2 × 10^5 per well were treated with compound **6e** at the IC_{50} (μM) for 48 h, then the cells were harvested and stained with Annexin V-FITC/ PI dye for 15 min in the dark at 37 °C. The samples were immediately analyzed using *FACS Calibur* flow cytometer (Becton and Dickinson, Heidelberg, Germany).

S4.2.5. Histopathological studies of compound 6e

S4.2.5.1. Experimental Animals

S4.2.5.1.1. Animal management

Adult female Swiss albino mice weighing 20-25 g were obtained from Abo Rawash culture – Giza used throughout this study. The animals were housed in steel mesh cages (Port Said University) and maintained on a commercial pellet diet and water ad libitum for one week before starting the experiment as an acclimatization period. All procedures performed in this study were approved by the Ethical Committee of Plant protection research Centre, Dokki, Egypt.

S4.2.5.1.2. Ehrlich Ascites Carcinoma cell (EAC)

Ehrlich ascites carcinoma (EAC) cells were initially supplied from the National Cancer Institute, Cairo, Egypt (only for the first transplantation), and maintained in

the peritoneal cavity of female Swiss albino mice through serial intraperitoneal (I.P.) injection at 8 or 10 day intervals in our laboratory in an ascites form.

S4.2.5.2. Experimental design

A total number of 30 female Swiss albino mice weighing 20-25 g were divided into the following groups (10 mice for each group) as follows:

Group (1): normal Control group

Group (2): Positive Control (EAC bearing group): mice were injected with Ehrlich ascites carcinoma (EAC) at the concentration of (2.5 $\times 10^6$ cells/mice) by I.P. injection once at the first day.

Group (3): mice were injected I.P. with coumarin-acrylamide **6e** (100 mg/kg i.p.) dissolved in DMSO the day after EAC injection, then the compound was injected day after day for 10 days.

S4.3. Molecular docking study

AutoDock Vina was utilized for conducting molecular docking studies. The three-dimensional configuration of the tubulin complex was obtained from the Protein Data Bank under the identifier 1SA0. Specifically, the complex formed by chain B of the tubulin heterodimer with colchicine was chosen for the modeling task. Ligands were sketched and underwent energy minimization, while the protein preparation was carried out using Discovery Studio Suite (Version 5.2).
